# Correction: Ethnic inequalities in COVID-19 vaccine uptake and comparison to seasonal influenza vaccine uptake in Greater Manchester, UK: A cohort study

**DOI:** 10.1371/journal.pmed.1003982

**Published:** 2022-04-22

**Authors:** Ruth Elizabeth Watkinson, Richard Williams, Stephanie Gillibrand, Caroline Sanders, Matt Sutton

There is an error in the Funding Disclosure. The correct Funding Disclosure reads:

This work was funded by an internal grant from the University of Manchester. University of Manchester website: https://www.manchester.ac.uk/. CS and MS and the PCIE input were part-funded by the National Institute for Health Research (NIHR) Applied Research Collaboration Greater Manchester (award number: NIHR200174). MS is a NIHR Senior Investigator. CS was part-funded and RW was funded by the NIHR Greater Manchester Patient Safety Translational Research Centre (award number: PSTRC-2016-003). The views expressed are those of the authors and not necessarily those of the NIHR or the Department of Health and Social Care. The funders had no role in the study design, data collection and analysis, decision to publish, or preparation of the manuscript.
