# Supplementary material for: Ethnic inequalities in COVID-19 vaccine uptake and comparison to seasonal influenza vaccine uptake in Greater Manchester, UK: A cohort study
Source: PLoS Med. 2022 Mar 3;19(3):e1003932. doi: 10.1371/journal.pmed.1003932 (PMC8893324; doi:10.1371/journal.pmed.1003932)
Supplement: S1 Appendix — Fig A: Flow chart showing inclusion criteria and missing data. GM, Greater Manchester; GP, general practice; LSOA, Lower Layer Super Output Area. LSOAs are neighbourhood-level administrative boundaries containing approximately 1,000 residents. Fig B: log(−log[survival]) versus log(time) plots stratified by ethnic group for COVID-19 and influenza vaccine uptake. Fig C: Kaplan–Meier failure curves indicating the cumulative probability of vaccination over time for (A) COVID-19 and (B) influenza vaccines, stratified by COVID-19 vaccination eligibility group. Individuals were assigned to the highest priority vaccine eligibility group for which they were eligible. Priority proceeded from those aged 80+ years down the age brackets, with the high clinical risk group eligible at the same time as the age group 70–74 years, and the moderate clinical risk group eligible between the age groups 65–69 and 60–64 years, as indicated in the legend group order. Vaccine eligibility groups are mutually exclusive, such that, for example, an individual aged 80 with high clinical vulnerability was categorized in the age 80+ eligibility group, whereas an individual aged 65 with high clinical vulnerability was categorized in the high clinical risk eligibility group. Fig D: Associations between ethnic group and vaccine uptake by gender (results also in Table F). Hazard ratios with 95% confidence intervals from Cox proportional hazards models estimating time to vaccination across ethnic groups, adjusted by vaccine eligibility group, stratified by gender. (A) Male; (B) female. Fig E: Associations between ethnic group and COVID-19 vaccine uptake—sensitivity analysis adjusting by locality or income deprivation (results also in Table G). Hazard ratios with 95% confidence intervals from Cox proportional hazards models estimating time to COVID-19 vaccination across ethnic groups, adjusted by vaccine eligibility group, plus additional adjustment by GM locality (10 local authority areas) or income dom [file pmed.1003932.s001.pdf]

S1 – Appendix

Figure A - Flow chart showing inclusion criteria and missing data. Abbreviations: Greater Manchester (GM), General Practice (GP), Lower-layer Super Output Area (LSOA) (LSOAs are neighbourhood-level administrative boundaries containing approx. 1000 residents).

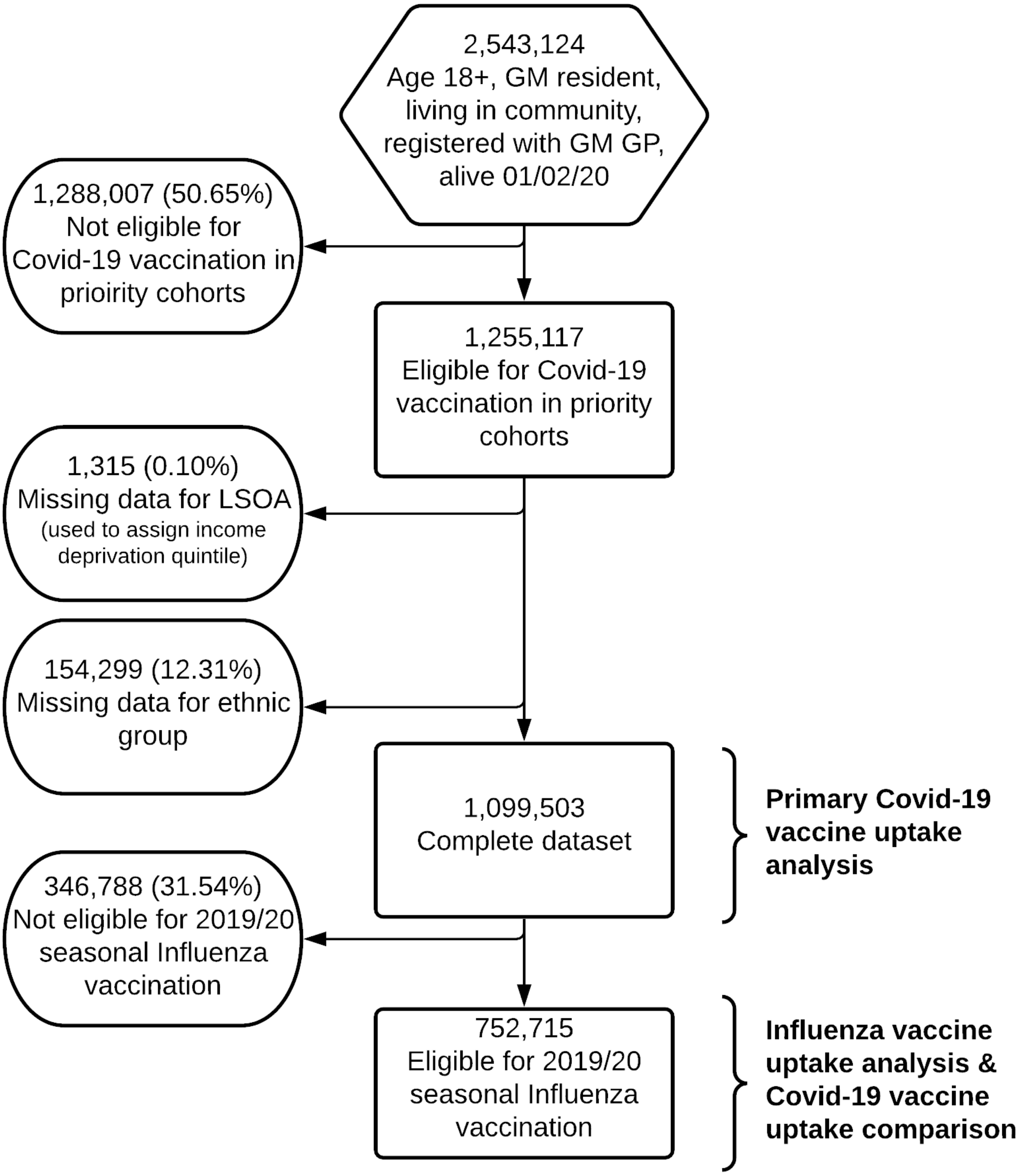

Figure B – log(-log(survival)) versus log(time) plots stratified by ethnic group for Covid-19 and Influenza vaccine uptake

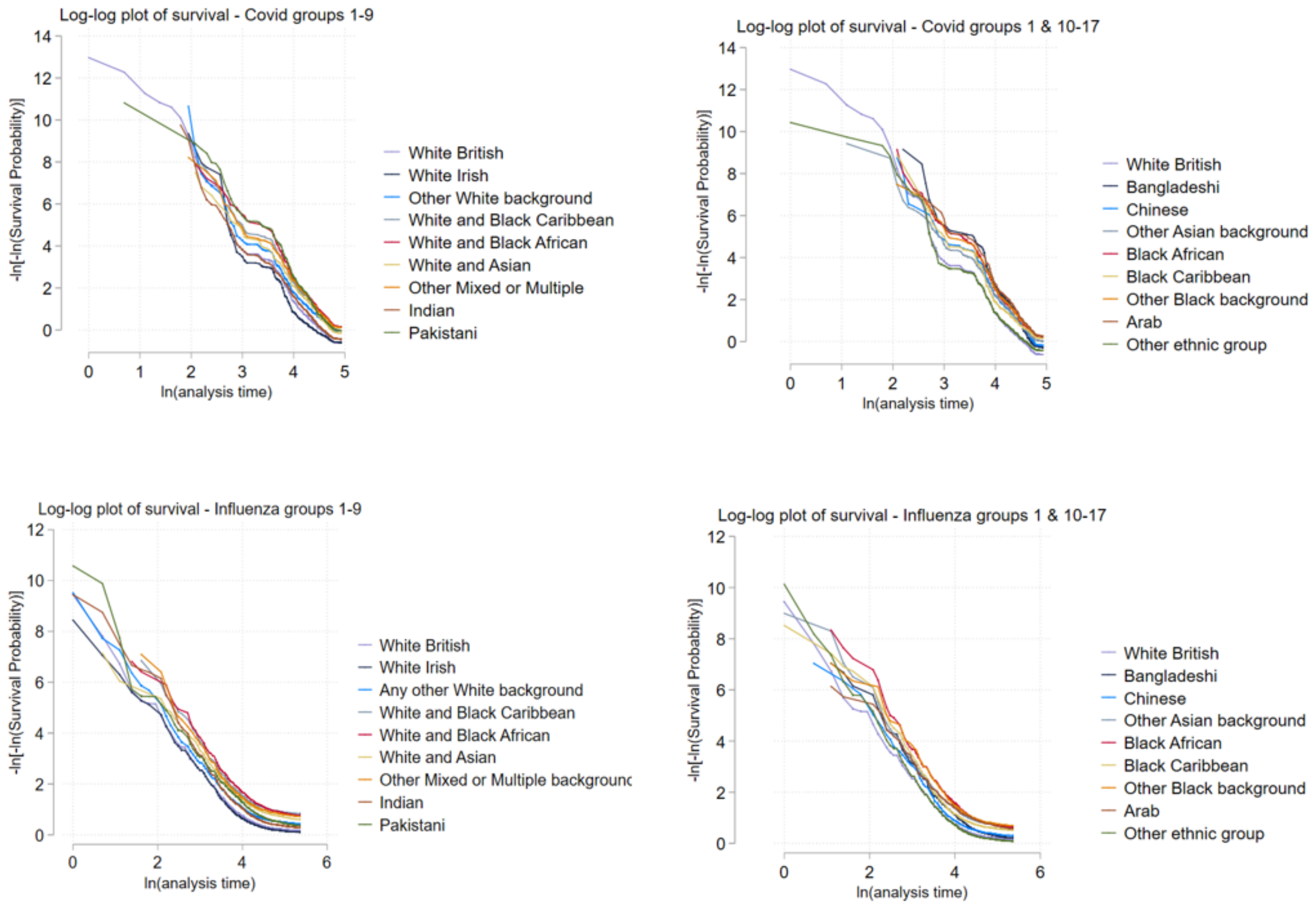

Figure C – Kaplan-Meier failure curves indicating the cumulative probability of vaccination over time for (A) Covid-19 and (B) Influenza vaccines, stratified by Covid-19 vaccination eligibility group. Individuals were assigned to the highest priority vaccine eligibility group for which they were eligible. Priority proceeded from those aged 80+ down the age brackets, with the high clinical risk group eligible at the same time as the 70-74 age group, and the moderate clinical risk group eligible between the 65-69 and 60-64 age groups, as indicated in the legend group order. Vaccine eligibility groups are mutually exclusive, such that (for example) an individual aged 80 with high clinical vulnerability would be in the 80+ age group, whereas an individual aged 65 with high clinical vulnerability would be in the high clinical vulnerability group.

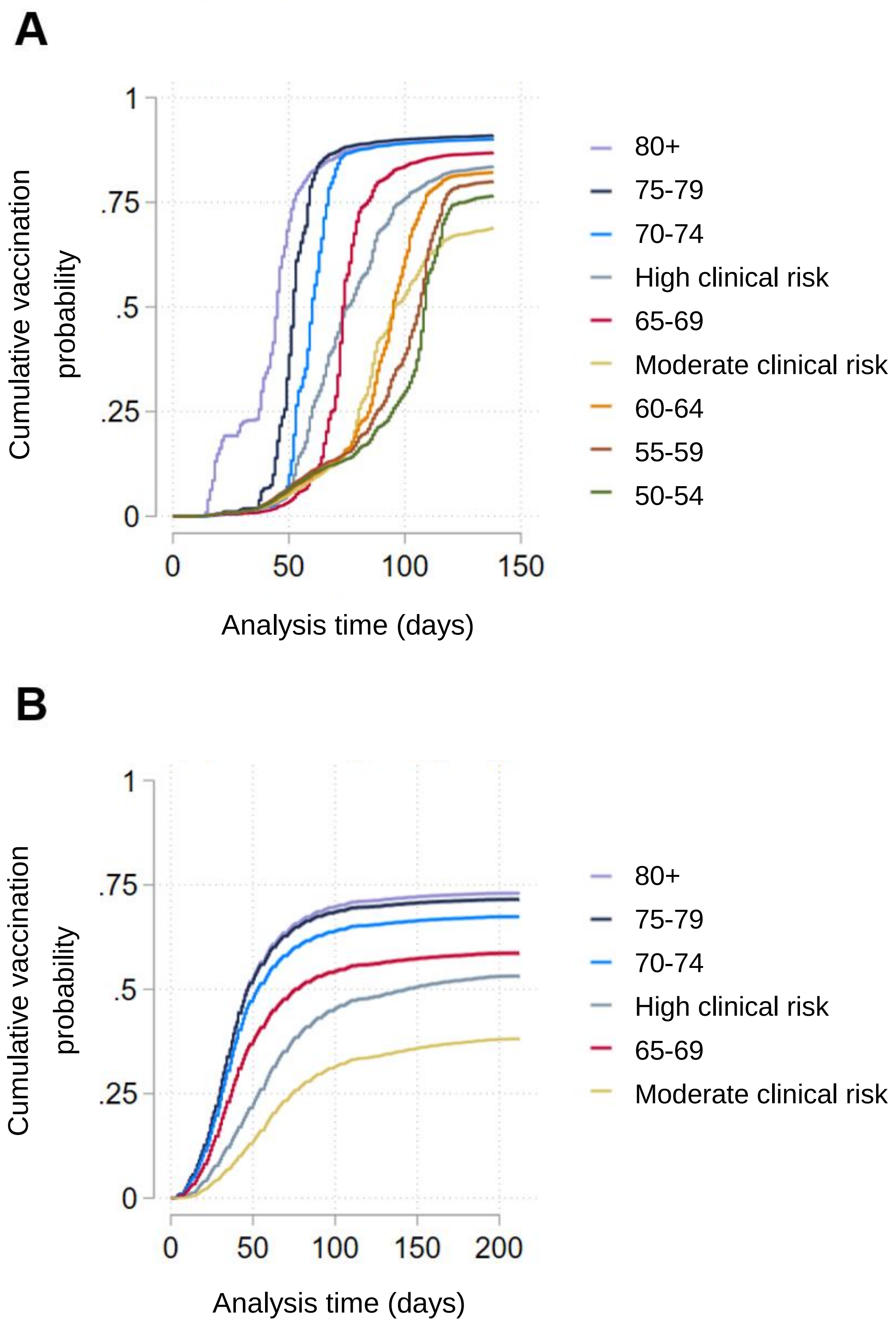

Table A – Vaccine uptake by population subgroup and vaccine type (percentage uptake and 95% ci)

	Covid-19 vaccine uptake 2020/2021		Influenza vaccine uptake 2019/20[1]	
	Percentage	95% ci	Percentage	95% ci
Ethnic group[2]				
White British	87.93	[87.86,88.00]	57.03	[56.90,57.16]
White Irish	86.40	[85.76,87.02]	59.07	[58.04,60.09]
Any other White background	69.97	[69.53,70.40]	47.32	[46.71,47.92]
White and Black Caribbean	64.31	[62.49,66.09]	35.13	[32.99,37.34]
White and Black African	67.05	[65.26,68.79]	37.10	[34.90,39.35]
White and Asian	77.35	[75.38,79.21]	41.70	[38.99,44.46]
Other Mixed or Multiple background	66.87	[65.34,68.37]	38.25	[36.32,40.22]
Indian	84.76	[84.22,85.29]	52.33	[51.45,53.22]
Pakistani	75.28	[74.90,75.66]	49.49	[48.99,49.99]
Bangladeshi	81.87	[81.08,82.64]	54.86	[53.75,55.96]
Chinese	75.47	[74.40,76.52]	52.12	[50.42,53.80]
Other Asian background	70.85	[70.04,71.64]	41.15	[40.07,42.23]
Black African	65.83	[65.15,66.51]	40.86	[40.00,41.73]
Black Caribbean	62.17	[61.04,63.28]	44.24	[42.84,45.64]
Other Black background	60.39	[58.78,61.99]	38.83	[36.83,40.86]
Arab	64.22	[61.67,66.69]	42.79	[39.63,46.02]
Any other ethnic group	82.84	[82.43,83.23]	59.54	[58.92,60.15]
Income deprivation[2]				
Least deprived	86.63	[86.47,86.78]	58.70	[58.41,58.98]
Q2	87.02	[86.86,87.17]	57.75	[57.47,58.03]
Q3	83.99	[83.83,84.15]	54.08	[53.80,54.35]
Q4	83.07	[82.91,83.22]	53.31	[53.06,53.56]
Most deprived	77.95	[77.83,78.07]	49.22	[49.04,49.40]
Gender				
Male	81.70	[81.60,81.80]	53.78	[53.62,53.94]
Female	83.24	[83.15,83.33]	52.93	[52.78,53.08]
Vaccine eligibility group				
High clinical risk	87.21	[87.02,87.40]	51.91	[51.63,52.20]
Moderate clinical risk	80.02	[79.87,80.17]	36.82	[36.64,37.00]
80+	87.47	[87.26,87.67]	70.18	[69.90,70.46]
75-79	89.34	[89.14,89.55]	69.00	[68.69,69.30]
70-74	88.23	[88.05,88.41]	64.47	[64.21,64.73]
65-69	84.64	[84.43,84.85]	55.39	[55.08,55.70]
60-64	78.87	[78.62,79.12]	N/A	-
55-59	78.34	[78.12,78.56]	N/A	-
50-54	76.20	[75.99,76.41]	N/A	-
Prior Influenza vaccination (2019/20)*				
Not vaccinated	74.64	[74.55,74.74]	N/A	-
Vaccinated	95.46	[95.40,95.52]	N/A	-

[1] Sample restricted to those eligible for Influenza vaccination

[2] More income-deprived quintiles and most minority ethnic groups have younger population age structures relative to less income-deprived groups and the White British group respectively, so % uptake figures presented in this table should be interpreted with caution as trends are substantially confounded by age

Table B – Associations between ethnic group and Covid-19 vaccine uptake (results also in Figure 1A) Hazard ratios with 95% confidence intervals from Cox proportional hazards models estimating time-to-Covid-19 vaccination across ethnic groups, stratified by vaccine eligibility group

	High clinical	Moderate clinical	80+	75-79	70-74	65-69	60-64	55-59	50-54
White British	Ref	Ref	Ref	Ref	Ref	Ref	Ref	Ref	Ref
	-	-	-	-	-	-	-	-	-
White Irish	0.908	0.975	0.940	1.096	1.062	0.923	0.876	0.872	0.817
	[0.829,0.995]	[0.920,1.033]	[0.900,0.982]	[1.041,1.155]	[1.006,1.120]	[0.870,0.980]	[0.806,0.952]	[0.804,0.946]	[0.756,0.882]
Other White background	0.578	0.581	0.844	0.796	0.749	0.621	0.542	0.553	0.522
	[0.554,0.602]	[0.565,0.596]	[0.810,0.879]	[0.758,0.834]	[0.720,0.779]	[0.596,0.647]	[0.520,0.566]	[0.533,0.573]	[0.505,0.539]
White and Black Caribbean	0.437	0.489	0.536	0.622	0.631	0.640	0.488	0.462	0.505
	[0.394,0.485]	[0.445,0.536]	[0.450,0.637]	[0.499,0.775]	[0.490,0.814]	[0.524,0.783]	[0.403,0.592]	[0.397,0.539]	[0.442,0.577]
White and Black African	0.468	0.525	0.387	0.471	0.513	0.455	0.481	0.428	0.504
	[0.430,0.510]	[0.477,0.577]	[0.269,0.557]	[0.302,0.732]	[0.399,0.659]	[0.358,0.578]	[0.393,0.589]	[0.364,0.504]	[0.448,0.566]
White and Asian	0.561	0.640	0.678	0.758	0.585	0.820	0.773	0.711	0.857
	[0.497,0.634]	[0.578,0.709]	[0.468,0.981]	[0.567,1.013]	[0.437,0.781]	[0.668,1.006]	[0.624,0.958]	[0.602,0.841]	[0.752,0.976]
Other Mixed or Multiple	0.454	0.518	0.511	0.562	0.505	0.690	0.477	0.526	0.528
	[0.415,0.498]	[0.479,0.561]	[0.411,0.635]	[0.442,0.716]	[0.417,0.612]	[0.585,0.813]	[0.403,0.565]	[0.467,0.593]	[0.476,0.586]
Indian	0.768	0.988	0.602	0.738	0.875	0.922	0.862	0.949	0.932
	[0.742,0.796]	[0.952,1.026]	[0.564,0.642]	[0.688,0.791]	[0.825,0.928]	[0.874,0.974]	[0.805,0.924]	[0.892,1.010]	[0.883,0.984]
Pakistani	0.485	0.626	0.461	0.484	0.564	0.608	0.512	0.522	0.557
	[0.475,0.495]	[0.614,0.638]	[0.440,0.482]	[0.457,0.513]	[0.538,0.591]	[0.586,0.632]	[0.484,0.541]	[0.499,0.547]	[0.539,0.576]
Bangladeshi	0.547	0.638	0.464	0.588	0.612	0.684	0.730	0.733	0.768
	[0.532,0.562]	[0.613,0.665]	[0.425,0.507]	[0.520,0.666]	[0.536,0.698]	[0.625,0.748]	[0.648,0.824]	[0.658,0.816]	[0.718,0.822]
Chinese	0.616	0.749	0.544	0.543	0.631	0.537	0.556	0.594	0.650
	[0.569,0.666]	[0.699,0.803]	[0.485,0.610]	[0.470,0.627]	[0.563,0.708]	[0.492,0.586]	[0.514,0.601]	[0.555,0.635]	[0.610,0.694]
Other Asian background	0.522	0.613	0.450	0.579	0.579	0.513	0.488	0.541	0.579
	[0.499,0.546]	[0.587,0.640]	[0.402,0.505]	[0.508,0.660]	[0.526,0.636]	[0.470,0.560]	[0.448,0.531]	[0.504,0.581]	[0.547,0.613]
Black African	0.448	0.468	0.276	0.291	0.329	0.386	0.401	0.435	0.471
	[0.435,0.462]	[0.449,0.489]	[0.241,0.317]	[0.251,0.338]	[0.295,0.366]	[0.349,0.426]	[0.367,0.439]	[0.408,0.465]	[0.450,0.494]
Black Caribbean	0.356	0.515	0.493	0.575	0.555	0.488	0.412	0.382	0.371
	[0.330,0.384]	[0.480,0.553]	[0.463,0.524]	[0.515,0.642]	[0.487,0.631]	[0.442,0.540]	[0.370,0.458]	[0.347,0.420]	[0.337,0.408]
Other Black background	0.398	0.456	0.409	0.378	0.411	0.462	0.477	0.419	0.419
	[0.367,0.432]	[0.415,0.500]	[0.330,0.506]	[0.286,0.500]	[0.325,0.521]	[0.383,0.558]	[0.396,0.575]	[0.368,0.477]	[0.374,0.470]
Arab	0.404	0.412	0.442	0.407	0.535	0.512	0.508	0.369	0.495
	[0.355,0.461]	[0.360,0.471]	[0.288,0.679]	[0.266,0.622]	[0.391,0.732]	[0.371,0.705]	[0.393,0.657]	[0.290,0.471]	[0.423,0.579]
Other ethnic group	0.679	0.839	1.087	1.064	0.965	0.881	0.804	0.721	0.695
	[0.655,0.703]	[0.817,0.861]	[1.050,1.124]	[1.027,1.101]	[0.935,0.997]	[0.849,0.914]	[0.766,0.844]	[0.691,0.752]	[0.668,0.723]
Observations	107879	251801	95177	79403	114478	101721	84850	114400	132840

Exponentiated coefficients; 95% confidence intervals in brackets

Table C – Vaccine uptake standardised to GM vaccine eligibility group structure. Estimated percentage uptake with 95% confidence intervals

	Covid-19 vaccine (all population)	Covid-19 vaccine (Influenza vaccine eligible population)	Influenza vaccine (Influenza vaccine eligible population)
White British	87.83 [87.76,87.90]	88.94 [88.86,89.02]	56.14 [56.01,56.26]
White Irish	84.11 [83.35,84.87]	86.51 [85.68,87.35]	53.53 [52.40,54.67]
Other White background	71.41 [70.99,71.83]	75.12 [74.61,75.63]	49.99 [49.41,50.57]
White and Black Caribbean	67.46 [65.49,69.43]	70.42 [67.98,72.86]	42.51 [39.72,45.31]
White and Black African	65.91 [63.37,68.45]	67.22 [63.81,70.63]	41.15 [37.58,44.72]
White and Asian	78.47 [76.20,80.73]	78.08 [75.15,81.01]	47.18 [43.60,50.76]
Other Mixed or Multiple	68.15 [66.40,69.90]	69.74 [67.48,72.00]	45.85 [43.41,48.29]
Indian	84.16 [83.58,84.73]	85.30 [84.63,85.96]	55.26 [54.36,56.15]
Pakistani	74.27 [73.82,74.73]	76.23 [75.70,76.76]	54.36 [53.76,54.96]
Bangladeshi	80.88 [79.83,81.92]	79.96 [78.75,81.18]	59.37 [57.94,60.81]
Chinese	76.12 [75.03,77.22]	77.85 [76.42,79.29]	52.79 [51.12,54.46]
Other Asian background	70.49 [69.59,71.40]	73.42 [72.27,74.57]	44.89 [43.61,46.17]
Black African	61.44 [60.46,62.42]	61.80 [60.50,63.10]	42.25 [40.94,43.56]
Black Caribbean	62.61 [61.46,63.76]	66.60 [65.20,68.01]	43.55 [42.08,45.02]
Other Black background	60.95 [58.96,62.94]	62.43 [59.81,65.05]	42.66 [39.96,45.35]
Arab	65.24 [62.06,68.41]	67.51 [63.34,71.68]	48.73 [44.40,53.06]
Other ethnic group	82.48 [82.07,82.88]	85.91 [85.48,86.34]	59.64 [59.06,60.23]

% vaccine uptake, 95% confidence intervals in brackets
Sample restricted to those alive at end of follow-up period

Table D – Associations between ethnic group and Influenza vaccine uptake (results also in Figure 1B)
Hazard ratios with 95% confidence intervals from Cox proportional hazards models estimating time-to-Influenza vaccination across ethnic groups, stratified by vaccine eligibility group

	High clinical	Moderate clinical	80+	75-79	70-74	65-69
White British	Ref<br>-	Ref<br>-	Ref<br>-	Ref<br>-	Ref<br>-	Ref<br>-
White Irish	0.932<br>[0.845,1.029]	0.980<br>[0.909,1.057]	0.862<br>[0.820,0.906]	0.920<br>[0.870,0.972]	0.880<br>[0.830,0.932]	0.888<br>[0.820,0.962]
Other White background	0.757<br>[0.721,0.794]	0.739<br>[0.714,0.765]	0.939<br>[0.902,0.978]	0.916<br>[0.872,0.961]	0.916<br>[0.879,0.955]	0.823<br>[0.783,0.864]
White & Black Caribbean	0.597<br>[0.518,0.688]	0.607<br>[0.536,0.688]	0.620<br>[0.505,0.762]	0.729<br>[0.539,0.984]	0.781<br>[0.599,1.018]	0.621<br>[0.462,0.834]
White and Black African	0.648<br>[0.579,0.726]	0.756<br>[0.671,0.852]	0.573<br>[0.380,0.864]	0.455<br>[0.278,0.746]	0.660<br>[0.499,0.875]	0.558<br>[0.408,0.763]
White and Asian	0.752<br>[0.642,0.882]	0.874<br>[0.768,0.995]	0.627<br>[0.405,0.970]	0.659<br>[0.484,0.898]	0.874<br>[0.652,1.171]	0.721<br>[0.547,0.950]
Other Mixed or Multiple	0.739<br>[0.660,0.826]	0.621<br>[0.557,0.693]	0.692<br>[0.539,0.889]	0.785<br>[0.597,1.032]	0.836<br>[0.694,1.006]	0.860<br>[0.691,1.072]
Indian	0.812<br>[0.773,0.852]	1.050<br>[1.003,1.100]	0.839<br>[0.783,0.898]	0.947<br>[0.875,1.025]	0.995<br>[0.933,1.062]	1.111<br>[1.043,1.183]
Pakistani	0.884<br>[0.861,0.907]	1.080<br>[1.055,1.106]	0.775<br>[0.737,0.815]	0.794<br>[0.746,0.844]	0.902<br>[0.859,0.948]	0.991<br>[0.949,1.035]
Bangladeshi	1.070<br>[1.023,1.119]	1.212<br>[1.151,1.277]	0.869<br>[0.786,0.960]	0.968<br>[0.845,1.108]	0.934<br>[0.821,1.062]	1.301<br>[1.172,1.444]
Chinese	0.820<br>[0.728,0.924]	1.035<br>[0.943,1.137]	0.885<br>[0.790,0.992]	0.798<br>[0.687,0.927]	0.852<br>[0.759,0.955]	0.926<br>[0.833,1.029]
Other Asian background	0.746<br>[0.699,0.795]	0.816<br>[0.771,0.864]	0.545<br>[0.481,0.618]	0.662<br>[0.574,0.763]	0.729<br>[0.655,0.810]	0.728<br>[0.656,0.807]
Black African	0.769<br>[0.740,0.798]	0.738<br>[0.700,0.778]	0.547<br>[0.477,0.628]	0.534<br>[0.463,0.617]	0.619<br>[0.553,0.692]	0.632<br>[0.560,0.714]
Black Caribbean	0.692<br>[0.633,0.757]	0.682<br>[0.622,0.749]	0.753<br>[0.702,0.808]	0.756<br>[0.672,0.851]	0.614<br>[0.532,0.709]	0.583<br>[0.507,0.671]
Other Black background	0.695<br>[0.628,0.770]	0.748<br>[0.667,0.838]	0.595<br>[0.469,0.756]	0.636<br>[0.484,0.836]	0.599<br>[0.462,0.778]	0.649<br>[0.510,0.824]
Arab	0.823<br>[0.697,0.972]	0.878<br>[0.754,1.022]	0.944<br>[0.629,1.417]	0.564<br>[0.315,1.009]	0.612<br>[0.430,0.872]	0.943<br>[0.676,1.314]
Other ethnic group	0.980<br>[0.939,1.023]	1.073<br>[1.037,1.110]	1.134<br>[1.095,1.174]	1.167<br>[1.120,1.216]	1.133<br>[1.092,1.176]	1.135<br>[1.083,1.191]
Observations	109199	253000	102371	81786	116595	89043

Exponentiated coefficients; 95% confidence intervals in brackets

Table E – Associations between ethnic group and vaccine uptake (results also in Figure 1C) Hazard ratios with 95% confidence intervals from Cox proportional hazards models estimating time-to-vaccination across ethnic groups, adjusted by vaccine eligibility group

	Covid-19 vaccine 2020/21	Influenza vaccine 2019/20	Covid-19 vaccine 2020/21 – excluding individuals who died during follow-up
White British	Ref	Ref	Ref
	-	-	-
White Irish	0.978	0.898	0.979
	[0.948,1.008]	[0.874,0.923]	[0.950,1.010]
Other White background	0.636	0.833	0.634
	[0.625,0.647]	[0.818,0.847]	[0.622,0.645]
White and Black Caribbean	0.496	0.626	0.495
	[0.466,0.528]	[0.581,0.675]	[0.465,0.527]
White and Black African	0.495	0.674	0.495
	[0.467,0.526]	[0.626,0.724]	[0.466,0.525]
White and Asian	0.627	0.788	0.627
	[0.585,0.672]	[0.725,0.857]	[0.585,0.672]
Other Mixed or Multiple	0.507	0.709	0.507
	[0.480,0.535]	[0.665,0.755]	[0.480,0.536]
Indian	0.788	0.943	0.788
	[0.770,0.806]	[0.921,0.966]	[0.770,0.806]
Pakistani	0.537	0.939	0.536
	[0.530,0.544]	[0.926,0.953]	[0.529,0.543]
Bangladeshi	0.577	1.082	0.576
	[0.564,0.591]	[1.051,1.113]	[0.562,0.589]
Chinese	0.575	0.897	0.573
	[0.549,0.603]	[0.856,0.941]	[0.547,0.601]
Other Asian background	0.540	0.736	0.539
	[0.524,0.556]	[0.711,0.762]	[0.523,0.555]
Black African	0.430	0.722	0.430
	[0.420,0.440]	[0.703,0.742]	[0.420,0.440]
Black Caribbean	0.435	0.695	0.434
	[0.418,0.454]	[0.667,0.724]	[0.416,0.452]
Other Black background	0.419	0.690	0.418
	[0.395,0.443]	[0.647,0.736]	[0.394,0.442]
Arab	0.434	0.824	0.434
	[0.399,0.473]	[0.748,0.907]	[0.399,0.473]
Other ethnic group	0.914	1.106	0.912
	[0.900,0.928]	[1.089,1.124]	[0.898,0.927]
High clinical	1.000	1.000	1.000
	[1.000,1.000]	[1.000,1.000]	[1.000,1.000]
Moderate clinical	0.529	0.630	0.528
	[0.526,0.533]	[0.624,0.636]	[0.525,0.532]
80+	2.535	1.874	2.563
	[2.501,2.569]	[1.854,1.894]	[2.528,2.598]
75-79	2.116	1.820	2.128
	[2.091,2.142]	[1.799,1.841]	[2.103,2.155]
70-74	1.525	1.596	1.529
	[1.511,1.539]	[1.579,1.613]	[1.515,1.543]
65-69	0.939	1.214	0.939
	[0.932,0.947]	[1.200,1.228]	[0.932,0.947]
Observations	737392	752494	728540

Exponentiated coefficients; 95% confidence intervals in brackets

Figure D – Associations between ethnic group and vaccine uptake by gender (results also in Table S6) Hazard ratios with 95% confidence intervals from Cox proportional hazards models estimating time-to-vaccination across ethnic groups, adjusted by vaccine eligibility group, stratified by gender. (A) Male, (B) Female.

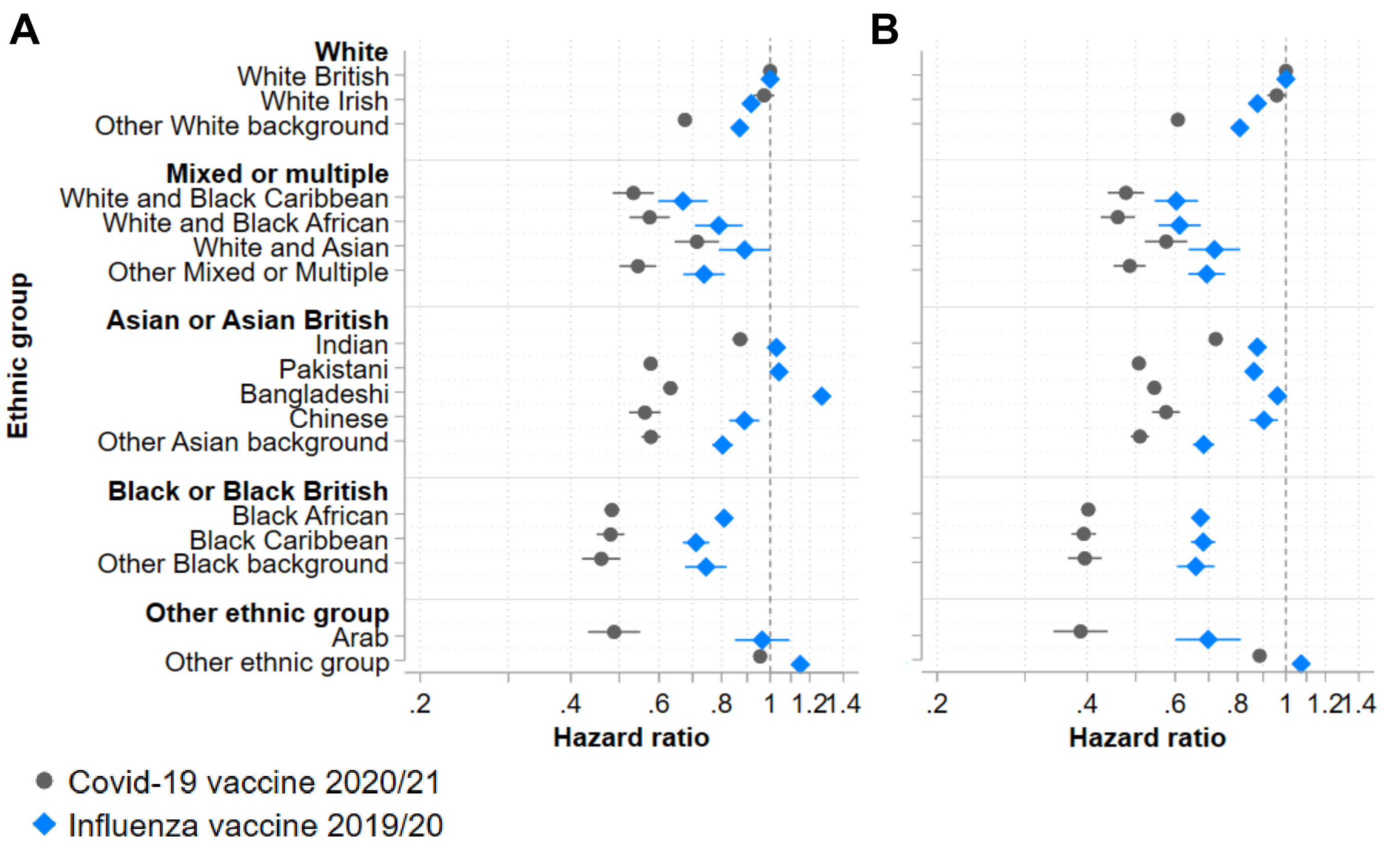

Table F – Associations between ethnic group and vaccine uptake by gender (results also in Figure S4) Hazard ratios with 95% confidence intervals from Cox proportional hazards models estimating time-to-vaccination across ethnic groups, adjusted by vaccine eligibility group, stratified by gender

	Male		Female	
	Covid-19 vaccine	Influenza vaccine	Covid-19 vaccine	Influenza vaccine
White British	Ref	Ref	Ref	Ref
	-	-	-	-
White Irish	0.972	0.916	0.958	0.877
	[0.927,1.019]	[0.880,0.953]	[0.918,1.000]	[0.844,0.911]
Other White background	0.676	0.869	0.607	0.808
	[0.658,0.694]	[0.847,0.892]	[0.593,0.622]	[0.789,0.828]
White and Black Caribbean	0.533	0.670	0.478	0.603
	[0.485,0.586]	[0.598,0.750]	[0.439,0.520]	[0.546,0.667]
White and Black African	0.575	0.790	0.460	0.612
	[0.524,0.631]	[0.707,0.882]	[0.425,0.498]	[0.555,0.675]
White and Asian	0.714	0.889	0.575	0.719
	[0.645,0.791]	[0.790,1.001]	[0.522,0.635]	[0.638,0.810]
Other Mixed or Multiple	0.544	0.738	0.486	0.694
	[0.500,0.592]	[0.670,0.812]	[0.452,0.524]	[0.638,0.756]
Indian	0.871	1.029	0.723	0.876
	[0.840,0.903]	[0.994,1.065]	[0.701,0.746]	[0.847,0.906]
Pakistani	0.577	1.041	0.507	0.863
	[0.566,0.589]	[1.021,1.063]	[0.498,0.516]	[0.845,0.880]
Bangladeshi	0.632	1.267	0.545	0.962
	[0.610,0.655]	[1.217,1.320]	[0.527,0.563]	[0.924,1.001]
Chinese	0.562	0.888	0.575	0.903
	[0.523,0.604]	[0.829,0.952]	[0.539,0.613]	[0.847,0.964]
Other Asian background	0.578	0.803	0.510	0.684
	[0.553,0.604]	[0.766,0.843]	[0.489,0.532]	[0.651,0.719]
Black African	0.483	0.809	0.402	0.674
	[0.466,0.501]	[0.778,0.842]	[0.389,0.415]	[0.650,0.699]
Black Caribbean	0.480	0.711	0.393	0.683
	[0.450,0.512]	[0.669,0.756]	[0.372,0.416]	[0.646,0.722]
Other Black background	0.460	0.744	0.395	0.660
	[0.421,0.503]	[0.676,0.819]	[0.366,0.427]	[0.605,0.720]
Arab	0.488	0.964	0.388	0.699
	[0.433,0.550]	[0.850,1.092]	[0.342,0.439]	[0.601,0.812]
Other ethnic group	0.955	1.149	0.886	1.073
	[0.933,0.978]	[1.122,1.176]	[0.866,0.906]	[1.050,1.098]
High clinical	Ref	Ref	Ref	Ref
	-	-	-	-
Mod. clinical	0.527	0.635	0.517	0.626
	[0.521,0.533]	[0.626,0.644]	[0.512,0.522]	[0.617,0.634]
80+	2.891	1.987	2.689	1.865
	[2.823,2.960]	[1.954,2.020]	[2.640,2.738]	[1.838,1.893]
75-79	2.241	1.830	2.201	1.875
	[2.196,2.286]	[1.798,1.862]	[2.164,2.239]	[1.845,1.905]
70-74	1.585	1.584	1.585	1.672
	[1.562,1.609]	[1.559,1.609]	[1.565,1.606]	[1.647,1.697]
65-69	0.928	1.172	0.984	1.312
	[0.916,0.940]	[1.152,1.193]	[0.973,0.995]	[1.291,1.334]
Observations	336543	344801	400340	407176

Exponentiated coefficients; 95% confidence intervals in brackets

Figure E – Associations between ethnic group and Covid-19 vaccine uptake – sensitivity analysis adjusting by locality or income deprivation (results also in Table S6) Hazard ratios with 95% confidence intervals from Cox proportional hazards models estimating time-to-Covid-19 vaccination across ethnic groups, adjusted by vaccine eligibility group, plus additional adjustment by GM locality (10 local authority areas) or income domain quintile

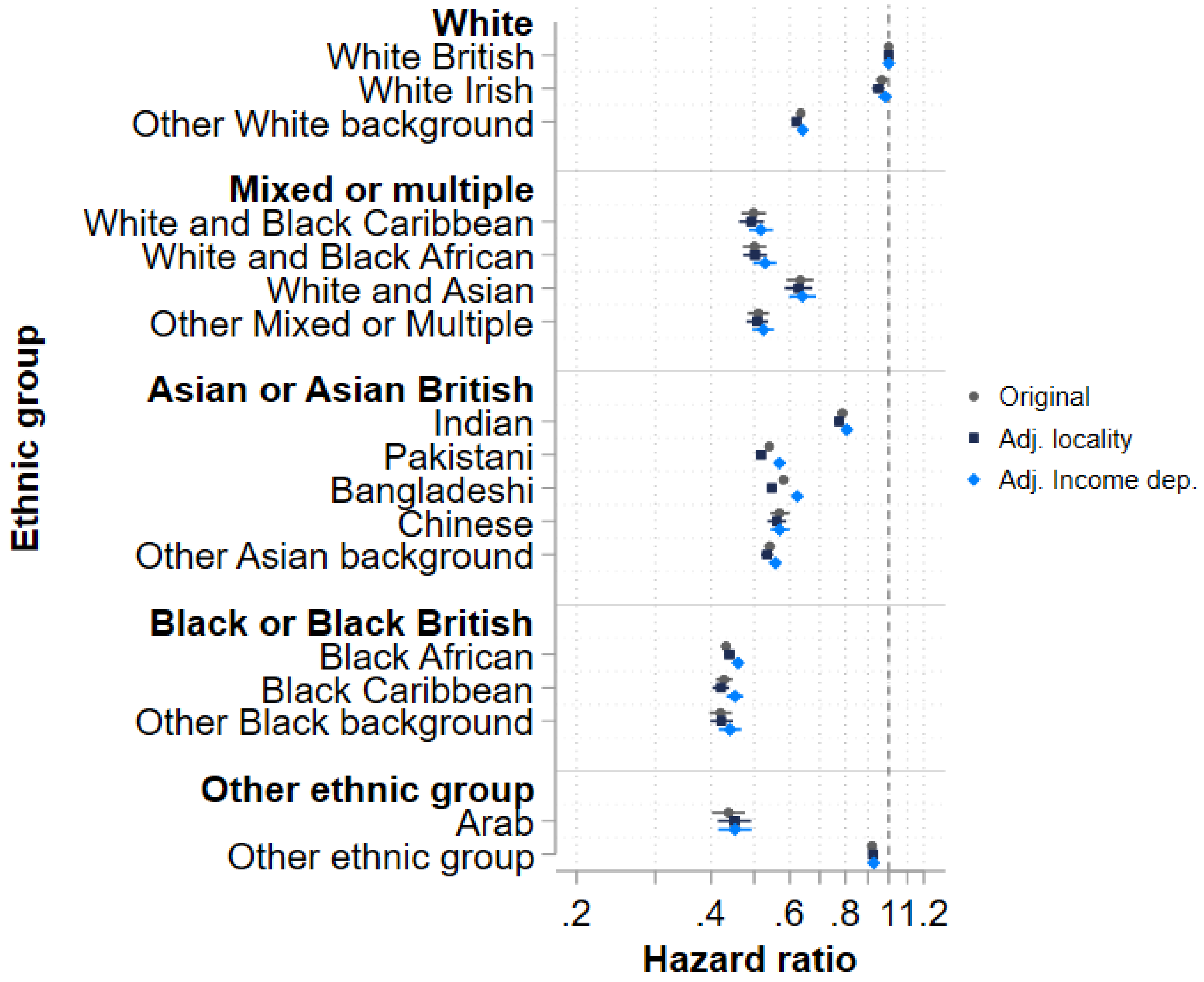

Table G – Associations between ethnic group and Covid-19 vaccine uptake – sensitivity analysis adjusting by locality or income deprivation (results also in Figure S4) Hazard ratios with 95% confidence intervals from Cox proportional hazards models estimating time-to-Covid-19 vaccination across ethnic groups, adjusted by vaccine eligibility group, plus additional adjustment by GM locality or income domain quintile

	Covid-19 vaccine (main analysis)	Covid-19 vaccine (adjusted by locality)	Covid-19 vaccine (adjusted by income deprivation quintile)
White British	Ref<br>-	Ref<br>-	Ref<br>-
White Irish	0.965<br>[0.935,0.996]	0.948<br>[0.918,0.979]	0.982<br>[0.952,1.014]
Other White background	0.635<br>[0.623,0.646]	0.622<br>[0.611,0.633]	0.642<br>[0.630,0.653]
White and Black Caribbean	0.498<br>[0.468,0.531]	0.493<br>[0.462,0.525]	0.517<br>[0.486,0.551]
White and Black African	0.501<br>[0.471,0.532]	0.502<br>[0.472,0.533]	0.529<br>[0.498,0.561]
White and Asian	0.634<br>[0.590,0.680]	0.629<br>[0.585,0.675]	0.641<br>[0.597,0.688]
Other Mixed or Multiple	0.511<br>[0.483,0.540]	0.508<br>[0.481,0.537]	0.524<br>[0.496,0.553]
Indian	0.788<br>[0.769,0.807]	0.774<br>[0.756,0.794]	0.806<br>[0.786,0.825]
Pakistani	0.540<br>[0.532,0.547]	0.517<br>[0.510,0.524]	0.570<br>[0.562,0.577]
Bangladeshi	0.581<br>[0.567,0.595]	0.547<br>[0.534,0.561]	0.624<br>[0.609,0.639]
Chinese	0.570<br>[0.543,0.598]	0.562<br>[0.535,0.590]	0.570<br>[0.544,0.599]
Other Asian background	0.541<br>[0.525,0.558]	0.534<br>[0.518,0.551]	0.558<br>[0.541,0.575]
Black African	0.432<br>[0.422,0.443]	0.439<br>[0.429,0.450]	0.460<br>[0.449,0.471]
Black Caribbean	0.428<br>[0.410,0.447]	0.421<br>[0.404,0.440]	0.453<br>[0.434,0.472]
Other Black background	0.420<br>[0.396,0.445]	0.422<br>[0.398,0.448]	0.441<br>[0.416,0.468]
Arab	0.438<br>[0.402,0.477]	0.452<br>[0.414,0.493]	0.453<br>[0.415,0.494]
Other ethnic group	0.917<br>[0.902,0.932]	0.924<br>[0.909,0.940]	0.926<br>[0.911,0.941]
High clinical	1.000<br>[1.000,1.000]	1.000<br>[1.000,1.000]	1.000<br>[1.000,1.000]
Mod. clinical	0.522<br>[0.519,0.526]	0.518<br>[0.514,0.522]	0.510<br>[0.506,0.514]
80+	2.776<br>[2.736,2.816]	2.814<br>[2.773,2.855]	2.669<br>[2.631,2.708]
75-79	2.225<br>[2.196,2.254]	2.271<br>[2.241,2.301]	2.131<br>[2.103,2.159]
70-74	1.588<br>[1.573,1.604]	1.601<br>[1.586,1.617]	1.525<br>[1.510,1.540]
65-69	0.959<br>[0.951,0.968]	0.957<br>[0.949,0.965]	0.922<br>[0.914,0.930]
Stockport		Ref<br>-	
Trafford		1.213<br>[1.195,1.231]	
Bury		1.323<br>[1.302,1.344]	

Wigan		0.778<br>[0.768,0.788]	
Tameside		1.119<br>[1.103,1.135]	
Rochdale		1.144<br>[1.129,1.161]	
Salford		0.984<br>[0.969,0.998]	
Bolton		0.999<br>[0.986,1.013]	
Oldham		1.101<br>[1.086,1.116]	
Manchester		0.953<br>[0.941,0.965]	
Least deprived			Ref<br>-
Q2			0.998<br>[0.986,1.010]
Q3			0.929<br>[0.919,0.940]
Q4			0.919<br>[0.909,0.929]
Most deprived			0.807<br>[0.799,0.815]
Observations	736898	736898	736898

Exponentiated coefficients; 95% confidence intervals in brackets

Table H – Associations between ethnic group and vaccine uptake by income deprivation (results also in Figure 2) Hazard ratios with 95% confidence intervals from Cox proportional hazards models estimating time-to-vaccination across ethnic groups, adjusted by vaccine eligibility group, stratified by income deprivation

	IMD income quintile 1 (least deprived)		IMD income quintile 5 (most deprived)	
	Covid-19 vaccine	Influenza vaccine	Covid-19 vaccine	Influenza vaccine
White British	Ref<br>-	Ref<br>-	Ref<br>-	Ref<br>-
White Irish	0.952<br>[0.869,1.043]	0.861<br>[0.794,0.935]	1.024<br>[0.975,1.076]	0.942<br>[0.901,0.984]
Other White background	0.669<br>[0.636,0.704]	0.927<br>[0.887,0.968]	0.569<br>[0.554,0.585]	0.734<br>[0.712,0.757]
White and Black Caribbean	0.647<br>[0.520,0.805]	0.662<br>[0.484,0.906]	0.469<br>[0.431,0.511]	0.656<br>[0.594,0.725]
White and Black African	0.754<br>[0.550,1.033]	0.880<br>[0.642,1.207]	0.475<br>[0.441,0.511]	0.670<br>[0.613,0.732]
White and Asian	0.763<br>[0.642,0.908]	0.944<br>[0.756,1.180]	0.573<br>[0.512,0.641]	0.779<br>[0.683,0.890]
Other Mixed or Multiple	0.696<br>[0.594,0.817]	0.765<br>[0.629,0.931]	0.460<br>[0.425,0.498]	0.721<br>[0.658,0.789]
Indian	0.802<br>[0.743,0.866]	0.830<br>[0.771,0.894]	0.811<br>[0.786,0.837]	1.010<br>[0.976,1.045]
Pakistani	0.674<br>[0.627,0.724]	0.783<br>[0.723,0.848]	0.547<br>[0.538,0.556]	1.001<br>[0.984,1.020]
Bangladeshi	0.596<br>[0.489,0.725]	0.974<br>[0.801,1.185]	0.610<br>[0.594,0.626]	1.148<br>[1.112,1.185]
Chinese	0.484<br>[0.430,0.545]	0.781<br>[0.693,0.881]	0.620<br>[0.575,0.668]	1.001<br>[0.928,1.081]
Other Asian background	0.592<br>[0.530,0.661]	0.744<br>[0.665,0.832]	0.522<br>[0.502,0.544]	0.746<br>[0.711,0.783]
Black African	0.430<br>[0.358,0.517]	0.661<br>[0.545,0.803]	0.456<br>[0.443,0.468]	0.750<br>[0.727,0.774]
Black Caribbean	0.635<br>[0.516,0.782]	0.914<br>[0.751,1.113]	0.438<br>[0.415,0.461]	0.725<br>[0.688,0.764]
Other Black background	0.435<br>[0.316,0.600]	0.792<br>[0.593,1.056]	0.407<br>[0.378,0.438]	0.706<br>[0.651,0.766]
Arab	0.323<br>[0.208,0.500]	0.660<br>[0.440,0.990]	0.461<br>[0.413,0.513]	0.855<br>[0.753,0.971]
Other ethnic group	1.085<br>[1.041,1.131]	1.194<br>[1.146,1.243]	0.769<br>[0.749,0.790]	1.009<br>[0.980,1.038]
High clinical	Ref<br>-	Ref<br>-	Ref<br>-	Ref<br>-
Mod. clinical	0.461<br>[0.451,0.472]	0.649<br>[0.628,0.671]	0.527<br>[0.521,0.533]	0.608<br>[0.600,0.617]
80+	2.373<br>[2.291,2.458]	1.972<br>[1.908,2.038]	2.267<br>[2.215,2.320]	1.781<br>[1.750,1.813]
75-79	1.774<br>[1.721,1.830]	1.916<br>[1.852,1.982]	2.052<br>[2.007,2.099]	1.681<br>[1.648,1.715]
70-74	1.263<br>[1.232,1.295]	1.728<br>[1.673,1.785]	1.546<br>[1.521,1.571]	1.459<br>[1.434,1.485]
65-69	0.779<br>[0.761,0.797]	1.349<br>[1.303,1.396]	0.962<br>[0.949,0.976]	1.107<br>[1.086,1.128]
Observations	100272	101966	278963	285088

Exponentiated coefficients; 95% confidence intervals in brackets

Table I – Associations between ethnic group and vaccine uptake by prior Influenza vaccine uptake (results also in Figure 3) Hazard ratios with 95% confidence intervals from Cox proportional hazards models estimating time-to-Covid-19 vaccination across ethnic groups, adjusted by vaccine eligibility group, stratified by prior Influenza vaccine uptake

	Had Influenza vaccine 2019/20	Did not have Influenza vaccine 2019/20
White British	Ref	Ref
	-	-
White Irish	1.002	1.013
	[0.957,1.048]	[0.970,1.059]
Other White background	0.589	0.657
	[0.574,0.603]	[0.638,0.678]
White and Black Caribbean	0.483	0.571
	[0.445,0.523]	[0.510,0.640]
White and Black African	0.496	0.528
	[0.458,0.538]	[0.480,0.581]
White and Asian	0.608	0.688
	[0.552,0.670]	[0.619,0.765]
Other Mixed or Multiple	0.498	0.553
	[0.463,0.535]	[0.506,0.605]
Indian	0.792	0.776
	[0.767,0.818]	[0.748,0.805]
Pakistani	0.535	0.494
	[0.525,0.545]	[0.484,0.504]
Bangladeshi	0.562	0.512
	[0.541,0.584]	[0.495,0.531]
Chinese	0.523	0.611
	[0.489,0.560]	[0.569,0.656]
Other Asian background	0.519	0.636
	[0.499,0.540]	[0.607,0.665]
Black African	0.427	0.435
	[0.414,0.442]	[0.419,0.452]
Black Caribbean	0.444	0.411
	[0.420,0.470]	[0.383,0.441]
Other Black background	0.389	0.486
	[0.359,0.421]	[0.444,0.532]
Arab	0.397	0.450
	[0.349,0.451]	[0.395,0.512]
Other ethnic group	0.796	0.914
	[0.775,0.817]	[0.893,0.934]
High clinical	Ref	Ref
	-	-
Moderate clinical	0.531	0.556
	[0.526,0.537]	[0.550,0.561]
80+	1.086	4.449
	[1.062,1.110]	[4.364,4.536]
75-79	1.184	3.124
	[1.158,1.210]	[3.075,3.175]
70-74	1.067	1.961
	[1.050,1.085]	[1.938,1.984]
65-69	0.847	0.999
	[0.835,0.859]	[0.989,1.010]
Observations	327440	409458

Exponentiated coefficients; 95% confidence intervals in brackets
