# Supplementary material for: Ethnic inequalities in COVID-19 vaccine uptake and comparison to seasonal influenza vaccine uptake in Greater Manchester, UK: A cohort study
Source: PLoS Med. 2022 Mar 3;19(3):e1003932. doi: 10.1371/journal.pmed.1003932 (PMC8893324; doi:10.1371/journal.pmed.1003932)
Supplement: S2 Missing Data for Ethnic Group — Table A: Missing ethnicity data by population subgroup (percentage missing and 95% CI). Table B: Census estimates of the percentage of individuals from each ethnic group by GM locality. Table C: Percentage of individuals in sample population from each ethnic group by GM locality, including missing data as an additional ethnic group category. Table D: Difference between sample population and census estimates (sample population estimate minus census estimate) of percentage of individuals from each ethnic group by GM locality, including missing data as an additional ethnic group category. Table E: Associations between ethnic group and vaccine uptake following partial recoding of missing ethnicity data (results also shown in Fig B). Hazard ratios with 95% confidence intervals from Cox proportional hazards models estimating time to vaccination across ethnic groups, adjusted by vaccine eligibility group, following partial recoding of missing ethnicity data to White British. Fig A: Histogram showing the distribution of percentage missing ethnic group data across LSOAs (neighbourhoods). Fig B: Associations between ethnic group and vaccine uptake following partial recoding of missing ethnicity data (results also shown in Table E). Hazard ratios with 95% confidence intervals from Cox proportional hazards models estimating time to vaccination across ethnic groups, adjusted by vaccine eligibility group, following partial recoding of missing ethnicity data to White British, compared to original ethnicity coding used in the main analysis. (A) COVID-19 vaccination; (B) 2019/2020 seasonal influenza vaccination. (PDF) [file pmed.1003932.s002.pdf]

S2 – Missing data for ethnic group

Given the relatively high level of missing data for ethnic group (12.31%), we conducted additional analysis to identify demographic characteristics of those with missing ethnicity data and to estimate whether missingness may have biased our estimates of ethnic inequalities in vaccine uptake. Missing ethnicity data may be missing at random, or differentially missing either because of differences between providers in data collection and completeness or individual refusal to provide ethnicity data information.

There was a higher percentage of missing ethnicity data amongst males, younger people, individuals who did not receive Influenza or Covid-19 vaccination, and those who remained alive during the analysis period (Table S10). Increasing neighbourhood-level income deprivation was associated with lower levels of missing ethnicity data, and there was substantial variation in missing ethnic group data across the 10 GM localities, ranging from 3.74% (95%c.i. 3.63-3.86) in Rochdale to 22.02% (95%c.i. 21.81-22.23) in Stockport (Table S10). There was also wide variability in missing ethnicity data at neighbourhood (LSOA) level (Figure S6), ranging from 0.54% (E01005591, Spotland and Falinge ward, Rochdale) to 44.79% (E01005906, Marple South ward, Stockport). Geographical clustering of missing ethnicity data at the neighbourhood and locality levels suggests missingness is likely due at least in part to differential local service provider collection, rather than individual refusal.

Estimates of the percentage of people from each ethnic group based on the 2011 UK Census are publicly available at locality level.[1] Table S11 shows these estimates and table S12 shows equivalent estimates from our dataset, including those with missing ethnicity data as an additional category. We compared these estimates, taking the simple difference between the two percentages for each ethnic group in each locality (Table S13). This indicated that for most localities (all except Rochdale, Oldham and Manchester) the White British group is overwhelmingly the most under-represented in our dataset compared to census estimates. This result is consistent with the over-representation of missing ethnicity data in areas that are less income-deprived, where White British residents are also over-represented.

Together, these results suggest a substantial proportion of missing ethnicity data may be due to poor service provider collection of ethnicity data in areas with a disproportionately high percentage of White British individuals. However, missing ethnicity data was also over-represented amongst those who did not receive vaccination. To test whether exclusion of those with missing ethnicity data may have biased our estimates of ethnic inequalities in vaccine uptake, we recoded missing ethnicity to White British for individuals in all boroughs except Rochdale, Oldham and Manchester. This included 110,270 individuals (71.47% of missing ethnicity data), leaving 3.51% missing in the total dataset. We then re-estimated ethnic inequalities in Covid-19 and Influenza vaccine uptake amongst those eligible for both vaccinations, adjusted by vaccine eligibility group as in the main analysis. Re-estimated ethnic inequalities in vaccine uptake were similar to those in the main analysis (Figure S7 (& main analysis Figure 1C) & Table S14 (& main analysis Table S5)), with any differences in hazard ratios less than 10%. Our conclusions are therefore robust to potential bias introduced by differential missing ethnicity data.

Additional references

1 Office for National Statistics: Population by ethnicity and region (2018) www.ethnicity-facts-figures.service.gov.uk/uk-population-by-ethnicity/national-and-regional-populations/regional-ethnic-diversity/latest/downloads/population-by-ethnicity-and-region.csv (accessed Sept 9, 2021)

Table A – Missing ethnicity data by population subgroups (percentage missing and 95% ci)

	Missing data for ethnic group	
	Percentage	95% ci
**Gender**		
Male	13.80	[13.72,13.89]
Female	10.92	[10.85,11.00]
**Locality[1]**		
Stockport	22.02	[21.81,22.23]
Trafford	17.21	[16.99,17.44]
Bury	12.04	[11.83,12.26]
Wigan	9.36	[9.22,9.51]
Tameside	9.85	[9.68,10.03]
Rochdale	3.74	[3.63,3.86]
Salford	12.77	[12.57,12.98]
Bolton	7.27	[7.13,7.41]
Oldham	6.25	[6.10,6.39]
Manchester	16.55	[16.39,16.71]
**Income deprivation**		
Least deprived	15.12	[14.95,15.28]
Q2	13.23	[13.08,13.38]
Q3	12.20	[12.06,12.35]
Q4	12.09	[11.96,12.22]
Most deprived	10.93	[10.84,11.02]
**Vaccine eligibility group**		
High clinical risk	7.55	[7.41,7.71]
Moderate clinical risk	9.33	[9.23,9.44]
80+	11.72	[11.54,11.91]
75-79	10.21	[10.02,10.41]
70-74	10.94	[10.77,11.11]
65-69	12.99	[12.80,13.18]
60-64	17.27	[17.04,17.51]
55-59	16.64	[16.45,16.84]
50-54	16.33	[16.15,16.52]
**Covid-19 vaccination (2020/21)**		
Not vaccinated	24.38	[24.21,24.56]
Vaccinated	9.48	[9.42,9.54]
**Influenza vaccination (2019/20)**		
Not vaccinated	15.87	[15.79,15.95]
Vaccinated	6.55	[6.48,6.62]
**Death during analysis period**		
No	12.36	[12.30,12.42]
Yes	10.12	[9.78,10.47]

[1] Localities ordered from least (Stockport) to most income-deprived

Figure A – Histogram showing the distribution of percentage missing ethnic group data across LSOAs (neighbourhoods)

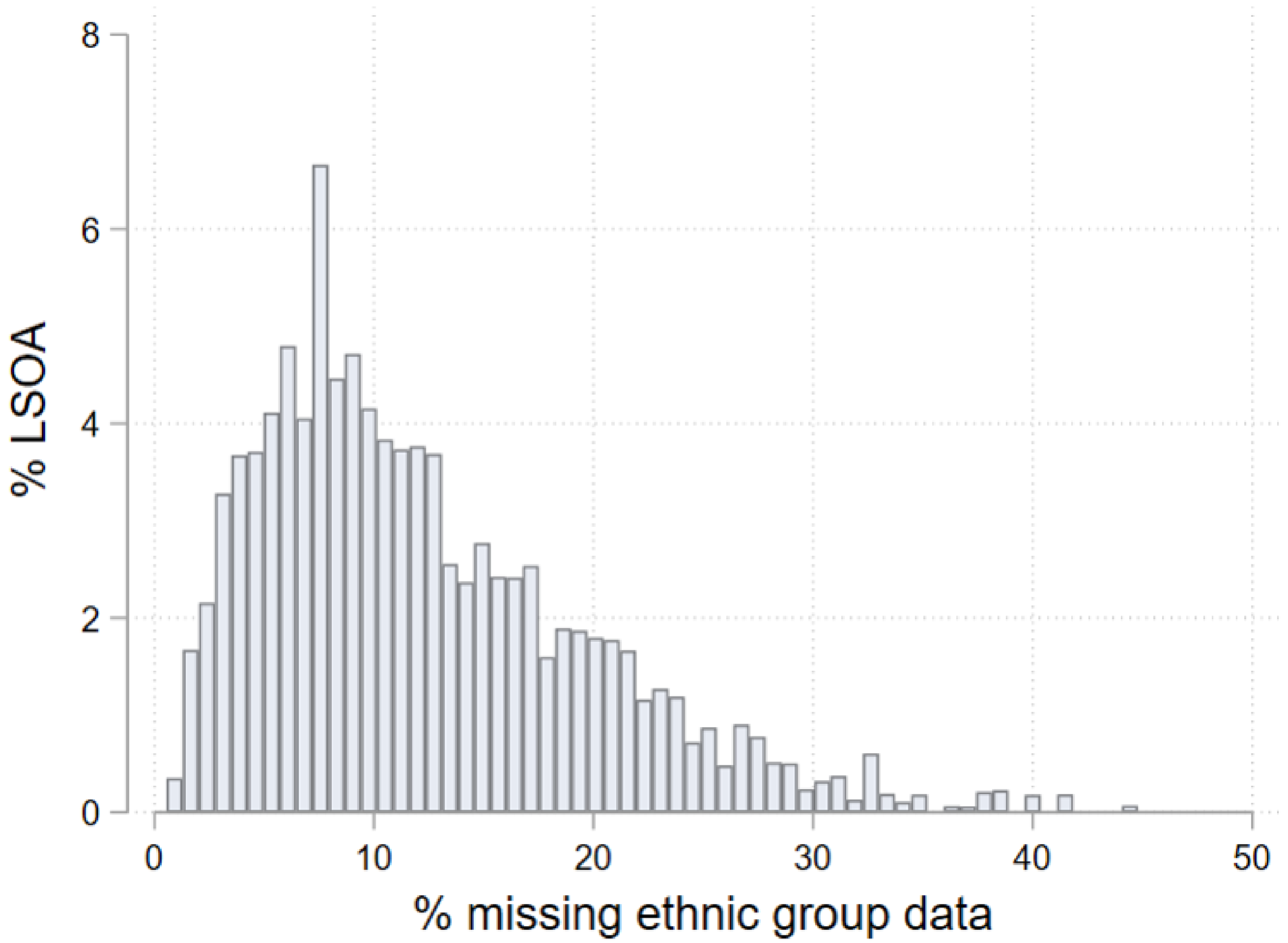

Table B – Census estimates of the percentage of individuals from each ethnic group by GM locality

	Stockport	Trafford	Bury	Wigan	Tameside	Rochdale	Salford	Bolton	Oldham	Manchester
White British	89.0	80.4	85.3	95.5	88.5	78.6	84.4	79.4	75.6	59.3
White Irish	1.4	2.2	1.3	0.5	0.7	1.0	1.2	0.6	0.7	2.4
Any other White background	1.7	2.9	2.5	1.3	1.7	2.0	4.5	1.9	1.3	5.0
White and Black Caribbean	0.6	1.2	0.7	0.3	0.6	0.5	0.7	0.6	0.9	1.8
White and Black African	0.3	0.3	0.2	0.1	0.2	0.3	0.5	0.2	0.2	0.9
White and Asian	0.5	0.7	0.5	0.2	0.4	0.6	0.4	0.7	0.5	1.0
Other Mixed or Multiple	0.4	0.5	0.3	0.2	0.2	0.3	0.4	0.3	0.2	1.0
Indian	1.0	2.8	0.7	0.3	1.7	0.5	1.1	7.8	0.7	2.3
Pakistani	2.4	3.1	4.9	0.2	2.2	10.5	0.8	4.3	10.1	8.5
Bangladeshi	0.2	0.2	0.2	0.0	2.0	2.1	0.3	0.2	7.3	1.3
Chinese	0.6	1.0	0.6	0.3	0.4	0.4	1.1	0.5	0.3	2.7
Other Asian background	0.7	0.9	0.9	0.3	0.3	1.4	0.8	1.1	0.8	2.3
Black African	0.3	0.8	0.6	0.4	0.5	1.0	2.3	1.2	0.7	5.1
Black Caribbean	0.3	1.7	0.3	0.1	0.2	0.1	0.3	0.2	0.4	1.9
Other Black background	0.1	0.4	0.1	0.0	0.1	0.2	0.2	0.2	0.1	1.6
Arab	0.3	0.6	0.3	0.1	0.1	0.1	0.6	0.3	0.1	1.9
Any other ethnic group	0.3	0.4	0.5	0.1	0.1	0.3	0.5	0.4	0.2	1.2
Refused or missing	-	-	-	-	-	-	-	-	-	-

Table C – Percentage of individuals in sample population from each ethnic group by GM locality, including missing data as an additional ethnic group category

	Stockport	Trafford	Bury	Wigan	Tameside	Rochdale	Salford	Bolton	Oldham	Manchester
White British	63.9	63.1	73.5	82.8	79.1	76.2	73.1	73.7	74.0	50.1
White Irish	0.7	1.6	1.0	0.4	0.5	0.7	1.0	0.5	0.6	1.9
Any other White background	6.3	2.6	5.1	1.8	2.5	3.3	3.7	2.8	2.4	3.9
White and Black Caribbean	0.1	0.4	0.1	0.0	0.1	0.1	0.1	0.1	0.3	0.6
White and Black African	0.1	0.2	0.1	0.1	0.1	0.2	0.3	0.1	0.2	0.6
White and Asian	0.1	0.2	0.1	0.1	0.1	0.1	0.1	0.1	0.1	0.3
Other Mixed or Multiple	0.3	0.3	0.2	0.1	0.2	0.3	0.3	0.2	0.2	0.7
Indian	0.8	1.9	0.6	0.2	1.4	0.4	0.6	5.5	0.6	1.4
Pakistani	1.7	2.4	3.6	0.1	1.8	9.1	0.7	3.9	7.5	8.2
Bangladeshi	0.1	0.1	0.1	0.0	1.2	1.1	0.2	0.1	4.3	0.9
Chinese	0.4	0.9	0.4	0.2	0.3	0.2	0.5	0.2	0.2	1.3
Other Asian background	0.7	1.1	1.1	0.3	0.5	0.7	0.9	1.0	0.7	2.3
Black African	0.3	0.7	0.5	0.4	0.6	1.3	1.7	1.3	0.9	5.1
Black Caribbean	0.2	1.3	0.3	0.1	0.2	0.1	0.2	0.2	0.4	2.1
Other Black background	0.1	0.4	0.1	0.1	0.2	0.2	0.3	0.2	0.2	0.8
Arab	0.1	0.1	0.0	0.0	0.0	0.0	0.1	0.1	0.0	0.4
Any other ethnic group	2.1	5.7	1.1	4.0	1.4	2.3	3.3	2.6	1.4	3.0
Refused or missing	22.0	17.2	12.0	9.4	9.9	3.7	12.8	7.3	6.3	16.6

Table D – Difference between sample population and census estimates (sample population – census) percentage of individuals from each ethnic group by GM locality, including missing data as an additional ethnic group category

	Stockport	Trafford	Bury	Wigan	Tameside	Rochdale	Salford	Bolton	Oldham	Manchester
White British	**-25.1**	**-17.3**	**-11.8**	**-12.7**	**-9.4**	-2.4	**-11.3**	**-5.7**	-1.6	-9.2
White Irish	-0.7	-0.6	-0.3	-0.1	-0.2	-0.3	-0.2	-0.1	-0.1	-0.5
Any other White background	4.6	-0.3	2.6	0.5	0.8	1.3	-0.8	0.9	1.1	-1.1
White and Black Caribbean	-0.5	-0.9	-0.6	-0.3	-0.5	-0.4	-0.6	-0.5	-0.7	-1.2
White and Black African	-0.2	-0.1	-0.1	0.0	-0.1	-0.1	-0.2	-0.1	0.0	-0.3
White and Asian	-0.4	-0.5	-0.4	-0.1	-0.3	-0.5	-0.3	-0.6	-0.4	-0.7
Other Mixed or Multiple	-0.1	-0.2	-0.1	-0.1	0.0	-0.1	-0.1	-0.1	0.0	-0.4
Indian	-0.2	-0.9	-0.1	-0.1	-0.3	-0.1	-0.5	-2.3	-0.1	-0.9
Pakistani	-0.7	-0.7	-1.3	-0.1	-0.4	-1.5	-0.1	-0.4	-2.6	-0.3
Bangladeshi	-0.1	-0.1	-0.1	0.0	-0.8	-1.0	-0.2	-0.1	-3.1	-0.4
Chinese	-0.2	-0.1	-0.2	-0.1	-0.1	-0.2	-0.6	-0.3	-0.1	-1.4
Other Asian background	0.0	0.2	0.2	0.0	0.2	-0.7	0.1	-0.1	-0.1	0.0
Black African	0.0	-0.1	-0.1	0.0	0.1	0.3	-0.6	0.1	0.2	0.0
Black Caribbean	-0.1	-0.5	0.0	0.0	0.0	0.0	-0.1	0.0	-0.1	0.2
Other Black background	0.0	-0.1	0.0	0.1	0.1	0.0	0.1	0.0	0.1	-0.8
Arab	-0.3	-0.5	-0.3	-0.1	-0.1	-0.1	-0.5	-0.2	-0.1	-1.5
Any other ethnic group	1.8	5.3	0.6	3.9	1.3	2.0	2.8	2.2	1.2	1.8
Refused or missing	**22.0**	**17.2**	**12.0**	**9.4**	**9.9**	3.7	**12.8**	**7.3**	6.3	16.6

Figure B – Associations between ethnic group and vaccine uptake following partial recoding of missing ethnicity data (results also shown in Table S13) Hazard ratios with 95% confidence intervals from Cox proportional hazards models estimating time-to-vaccination across ethnic groups, adjusted by vaccine eligibility group, following partial recoding of missing ethnicity data to White British compared to original ethnicity coding used in the main analysis. (A) Covid-19 vaccination. (B) 2019/20 seasonal influenza vaccination

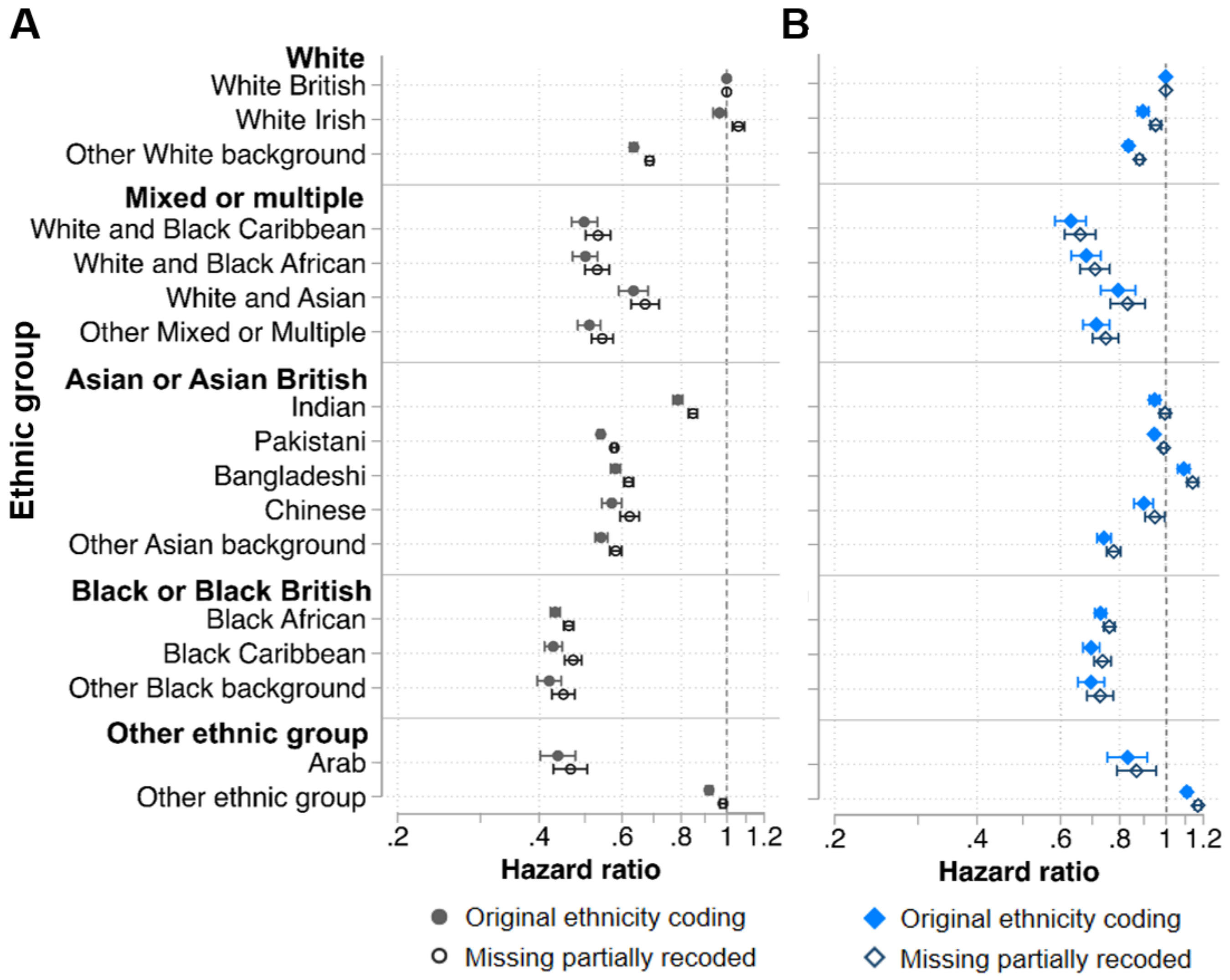

Table E - Associations between ethnic group and vaccine uptake following partial recoding of missing ethnicity data (results also shown in Figure S6) Hazard ratios with 95% confidence intervals from Cox proportional hazards models estimating time-to-vaccination across ethnic groups, adjusted by vaccine eligibility group, following partial recoding of missing ethnicity data to White British

	Covid-19 vaccine uptake 2020/21	Influenza vaccine uptake 2019/20
White British	Ref	Ref
	-	-
White Irish	1.059	0.952
	[1.027,1.091]	[0.927,0.979]
Other White background	0.686	0.880
	[0.674,0.698]	[0.865,0.895]
White and Black Caribbean	0.533	0.660
	[0.501,0.566]	[0.612,0.711]
White and Black African	0.531	0.709
	[0.500,0.563]	[0.659,0.762]
White and Asian	0.671	0.830
	[0.626,0.719]	[0.764,0.903]
Other Mixed or Multiple	0.544	0.747
	[0.516,0.574]	[0.701,0.795]
Indian	0.848	0.996
	[0.829,0.867]	[0.972,1.020]
Pakistani	0.577	0.990
	[0.570,0.584]	[0.976,1.004]
Bangladeshi	0.619	1.139
	[0.605,0.633]	[1.107,1.172]
Chinese	0.622	0.948
	[0.594,0.651]	[0.905,0.994]
Other Asian background	0.581	0.776
	[0.564,0.598]	[0.750,0.803]
Black African	0.462	0.760
	[0.451,0.473]	[0.740,0.781]
Black Caribbean	0.472	0.735
	[0.453,0.492]	[0.706,0.766]
Other Black background	0.450	0.727
	[0.425,0.476]	[0.682,0.775]
Arab	0.466	0.867
	[0.428,0.506]	[0.788,0.955]
Other ethnic group	0.982	1.169
	[0.967,0.997]	[1.150,1.188]
High clinical	1.000	1.000
	[1.000,1.000]	[1.000,1.000]
Mod. clinical	0.533	0.631
	[0.529,0.536]	[0.625,0.637]
80+	2.430	1.840
	[2.397,2.463]	[1.821,1.860]
75-79	2.066	1.803
	[2.040,2.091]	[1.782,1.824]
70-74	1.491	1.579
	[1.477,1.504]	[1.562,1.596]
65-69	0.925	1.202
	[0.917,0.933]	[1.188,1.216]
Observations	797143	813587

Exponentiated coefficients; 95% confidence intervals in brackets
