# Supplementary material for: Ethnic inequalities in COVID-19 vaccine uptake and comparison to seasonal influenza vaccine uptake in Greater Manchester, UK: A cohort study
Source: PLoS Med. 2022 Mar 3;19(3):e1003932. doi: 10.1371/journal.pmed.1003932 (PMC8893324; doi:10.1371/journal.pmed.1003932)
Supplement: S3 Analysis Plan — (PDF) [file pmed.1003932.s003.pdf]

Supporting information – Analysis plan

Changes from the analysis plan

As outlined in the methods section of the main paper, themes and priorities raised in our early Public and Community Involvement and Engagement (PCIE) sessions led to our focus on ethnic inequalities in vaccine uptake (as opposed to inequalities in vaccine eligibility), hence the focus on inequalities in vaccine uptake between ethnic groups, part of our original objective 5. The PICE discussions also prompted comparison with seasonal Influenza vaccine uptake, which was not part of the original objective 5 analysis plan. Given this comparative addition to the analysis, we decided to focus on inequalities between ethnic groups, as this is the sociodemographic factor with the largest inequalities in Covid-19 vaccine uptake. Although it is beyond the scope of this paper, we intend to do additional analysis as outlines in objective 5 to estimate additional types of inequalities.

Following further discussion within the research team we decided not to use the 80% coverage point for analysis, and instead modelled inequalities in vaccine uptake over the whole follow-up period to prevent potential biases that could be introduced by setting a threshold.

Original analysis plan (Copied verbatim from original project proposal in application to use the GMCR dataset)

Research Question

How equitable is the UK COVID-19 vaccination strategy and rollout in terms of vaccine access and receipt relative to risk of COVID-19 hospitalisation and mortality, with respect to geographical area, income deprivation and ethnicity?

Motivation

Following the development and approval of several highly effective vaccines to prevent COVID-19, establishing prioritisation criteria for distribution of the initially scarce supply is necessary. The UK government is distributing the vaccine free at the point of service via the NHS, which operates under the guiding principle of equal access for equal need. Given the widespread transmission and current high levels of COVID-19 mortality, the cost of delayed vaccination could be high for individuals. There are also costs to society if vaccines are not distributed to those with the highest risk of hospitalisation first, as the NHS is currently under severe pressure due to the volume of COVID-19 patients and economically damaging lockdown measures have been enacted to reduce further viral spread. For the purposes of this study, we are defining ‘equal access’ as being in the same vaccination priority group, and ‘equal need’ as the same relative risk of (1) COVID-19 mortality or (2) COVID-19 hospitalisation. The overarching goal of the initial rapid phase of this project (objectives 1-4 and PCIE) is to study whether the vaccination strategy achieves equal access for equal need in terms of geographical area, income deprivation and ethnicity. As vaccine roll-out progresses, we will also investigate emerging inequalities in vaccine uptake across socio-demographic groups (objective 5).

Proposed study outcomes

1. Estimation of how income deprivation and ethnicity interact with age to moderate risk of COVID-19 mortality and hospitalisation
2. Estimation of geographical, income and ethnic inequalities within Greater Manchester (GM) arising from the UK COVID vaccination strategy
3. Initial exploration of public attitudes to the equity implications of the UK COVID vaccination strategy
4. Estimation of geographical, income and ethnic inequalities in COVID vaccination uptake within GM

Outcome measure(s): Objectives 1-3: COVID-19 mortality (ONS definition – death within 28 days of positive COVID-19 test) within the study period. COVID-19 hospitalisation as secondary outcome.

Objective 4: Ratio of number eligible for vaccination at several phases of vaccine prioritisation against (1) total COVID-19 mortality and (2) total hospitalisations for COVID-19 for each MSOA in GM

Objective 5: Receipt of COVID vaccination (1st dose). Secondary outcomes - second dose of vaccine, vaccine type, vaccine offered but declined.

Exposure variables: Age, sex, ethnicity, index of multiple deprivation (IMD) income domain composite IMD, LSOA (to assign LSOA-level IMD), MSOA (can derive from LSOA if needed), GM locality, long-term conditions and medications relevant to COVID risk and/or COVID risk groups (using COVID moderate risk and high risk SNOMED codes if well populated in GMCR, but alternatively using the published criteria (using long term condition and medication SNOMED codes) to derive these groups), resident in care home, distance to nearest COVID vaccination hub from residential postcode (or LSOA centroid if needed), distance to site of actual receipt of COVID vaccine from residential postcode (or LSOA centroid if needed).

Objective 4 also MSOA-level weighted average IMD income domain and proportion of residents from ethnic minority groups

Study objectives

(1) Identify how age and income deprivation interact to moderate risk of COVID-19 mortality and hospitalisation within Greater Manchester

Previous work has demonstrated that, after adjustment for age, people living in more deprived areas have statistically significantly higher risk of COVID mortality and hospitalisation than those living in more affluent areas. However, there is currently no information about how this relative risk varies across the age risk gradient, so we aim to estimate this for the GM population.

Specifically, we will model Cox proportional hazards for the outcomes of (1) COVID-19 mortality and (2) COVID-19 hospitalisation. We will fit age (likely as an exponential function) interacted with IMD quintile, repeating analysis with adjustment and stratification by sex. We will also generate iso-risk contours from model estimates (i.e. two-way plots of combinations of deprivation and age that generate the same expected risk).

Outcomes will be unconditional on SARS-CoV-2 infection (i.e. outcomes per population rather than per those testing positive) due to wide differences in community testing availability over the study period and incomplete testing data in the GMCR.

We will not adjust for additional covariates as we want to capture total relative risks between IMD quintiles across the age distribution. With the exceptions of health and social care staff and care home residents, the vaccination strategy prioritisation first incorporates risk only by age, followed by defined clinical vulnerability after certain age groups. We therefore want to condition only on age in our initial analysis (+/- stratification and adjustment by sex), in order to estimate inequalities between IMD groups in the most relevant context. We are also conscious that the policy implications of this work may suggest additional factors should be considered in assignment of vaccine priority groups. For this to be actionable, it will be important that groups are as simply defined as possible, and that they rely on readily available and reliable data, such as IMD quintile. Analysis will be extended to incorporate clinical vulnerability to COVID in objective 3.

(2) Identify how age and ethnicity interact to moderate risk of COVID-19 mortality and hospitalisation within Greater Manchester

Existing studies have found elevated age-adjusted risk of COVID mortality and hospitalisation amongst ethnic minority groups relative to the White population in England. However, it is currently unclear whether or how this elevated risk varies across the age distribution.

We will use the same analytical approach as in objective 1 to estimate relative risk of COVID-19 mortality and hospitalisation across the age distribution for each ethnic group. Due to uncertainty about statistical power and missing data, we anticipate using broad categories (e.g. White, Black, Asian, Other, Missing; or White British, All minority ethnic groups, Missing). Whichever specified categories are used, we will keep ‘missing’ as a separate category given that we anticipate substantial missing data which may be differential with respect to actual ethnicity and/or other variables of interest.

(3) Using the vaccination strategy phase 1 prioritisation groups, estimate inequalities in vaccine access per relative risk of COVID mortality and hospitalisation by income deprivation and ethnicity

Extending the analysis in objectives 1 and 2 to model inequalities between more explicitly policy-relevant groups, we will assign a variable for the 9 vaccination phase 1 priority groups and the rest of the population: (1) Care home residents (if possible using GMCR) (2) 80+ (3) 75-79 (4) 70-74 and high clinical vulnerability (5) 65-69 (6) moderate clinical vulnerability (7) 60-64 (8) 55-59 (9) 50-54 (10) rest of population 16-49.

We will then estimate inequalities in vaccination access (defined as prioritisation group) per relative risk of (1) COVID mortality and (2) COVID hospitalisation across IMD quintiles by fitting the vaccination priority groups interacted with IMD quintile in a Cox proportional hazards model (+/- adjustment and stratification by

sex). We will interpret the IMD hazard ratios within each priority group as the degree of inequality by income deprivation.

We will then use the hazard ratios generated to rank the 50 priority group-x-IMD quintile subgroups in order of decreasing risk and split these into 10 alternative priority groupings (e.g. an alternative priority group could be age 75-79 from IMD Q1-3 + 65-70 from IMD Q4-5). We will estimate predicted ethnic inequalities using (a) the original priority groups and (b) the alternative groups identified, which take risk by IMD quintile into account. To do this, we will fit a Cox proportional hazards model for (1) COVID mortality and (2) COVID hospitalisation with (a) original priority groups and (b) alternative priority groups interacted with ethnicity.

We will interpret the ethnicity hazard ratios within each group as the degree of ethnic inequality, and will compare results from models (a) and (b) to assess whether incorporating income deprivation into the prioritisation strategy could reduce ethnic inequalities.

The rationale is that given the incomplete recording of ethnicity in primary care records, ethnicity is unlikely to be considered a feasible criterion for vaccine prioritisation. However, given the strong overrepresentation of people belonging to ethnic minority groups in more income deprived areas, incorporating IMD into the prioritisation strategy could reduce inequalities by ethnicity.

(4) Compare the distribution of vaccine eligibility with the distribution of COVID mortality and hospitalisation by geographical area, income deprivation and ethnicity

An alternative way to conceptualise inequalities in the vaccination strategy is to analyse at the level of population subgroups, and ask for each subgroup whether the proportion of people eligible for vaccination relative to subgroup 'need' (COVID mortality or hospitalisation rate) is equal at any given phase of the vaccination plan. Greater Manchester, areas of income deprivation, and ethnic minority groups all have younger population age structures than the total population in England. Prioritisation primarily by age suggests that as each vaccine priority group is completed, a smaller proportion of people within such populations will become eligible for vaccination than in demographic groups with older population structures. However, this distributional effect may run counter to the relative burden of COVID mortality across population groups.

We will calculate the ratio of number eligible for vaccination at several phases of vaccine prioritisation against (1) total COVID-19 mortality and (2) total hospitalisations for COVID-19 for each MSOA in GM. If distribution at MSOA level is equitable in terms of equal access for equal need, we would expect this ratio to be approximately constant across MSOAs, with only stochastic deviations. To test this, we will then regress MSOA ratios against the following variables in turn: (a) IMD quintile, (b) proportion of ethnic minority residents, (c) GM locality, interpreting any associations as evidence of inequitable distribution.

GM has 345 MSOAs and average MSOA population is 8300 (range ~5000-15000). Using the England average, expected COVID-19 mortality per 8300 is approximately 12, which is a likely underestimate for GM. MSOA income deprivation can be estimated as weighted population average derived from IMD income domain of component LSOAs.

Finally, we will compute and present ratios as above for larger population subgroups, comparing: (a) IMD quintiles within GM, (b) ethnic groups within GM, (c) each locality within GM, (d) GM compared to the total population in England.

(Total England population estimates for each priority group and COVID deaths by date are publicly available, so can be used as a reference for comparison (d), England-wide hospitalisation data will be used if possible.)

(5) Estimate inequalities in COVID vaccination uptake by GM locality, IMD quintile, ethnic group and gender

There are widely reported concerns about COVID vaccine hesitancy, both in the total population and particularly amongst some demographic groups including more income-deprived communities and some ethnic groups. As the vaccine roll-out progresses, we will monitor uptake across demographic groups and estimate emerging inequalities.

We will first identify the dynamics and coverage of COVID vaccination for each priority group, using the eligibility variable assigned in objective 3 to plot percent coverage over time. From this, we will identify the approximate point of the coverage plateau for each priority group. We will then analyse vaccination rates across socio-demographic groups up to the time point of 80% of plateau level using Cox proportional hazards regression models (using the 80% point because we assume the tail in coverage may be less predictable to model and different groups may plateau at different coverage rates). We will then use logistic regression to estimate the relative odds of remaining unvaccinated despite being in an eligible group after a short time lag after the point of plateau for that group.

For both the rates of vaccination and the odds of remaining unvaccinated, we will test for associations with the following variables in turn and in interacted combinations: (a) IMD quintile, (b) ethnic group, (c) gender, (d) GM locality. Analysis of rates up to 80% of total coverage may reveal supply-side inequalities or vaccine hesitancy within priority group in terms of timing of actual vaccine receipt, whereas analysis of those remaining unvaccinated likely reflects vaccine hesitancy translating into refusal.

We aim to use all 18 UK Census ethnic groups (plus 'missing' as an additional category) in this analysis, as the number of outcomes (vaccinations) is anticipated to be orders of magnitude higher than outcomes (mortality or hospitalisation) in the previous analyses.

Importantly, interactions between GM locality and the other exposures could suggest that inequalities may have been mitigated by work in some local areas. Analysis stratified by GM locality could then form the basis for further mixed-methods research working with people involved in vaccination roll out in some of the localities.

If possible, we will extend analysis to also incorporate distance from residential postcode (or LSOA centroid) to nearest COVID vaccination hub and to site of actual vaccine receipt to assess whether (1) there are socio-demographic inequalities in distances to vaccination sites and (2) whether distance needed to travel is associated with likelihood of vaccination.

As more information becomes available about the relative efficacy of each vaccine, we may also extend analysis to model inequalities in which specific vaccine individuals receive.

If possible, we may also extend analysis to estimate QALYs saved due to COVID vaccination across different subgroups, but this is likely to require further information about relative effectiveness of each of the 3 vaccines and various dosing regimens and it is not clear at what point this information may be available.

PCIE plan

Alongside statistical analysis, we intend to conduct Public and Community Involvement and Engagement (PCIE) work, starting with discussion with the established Health Innovation Manchester Forum and ARC-GM PPIE panel. We will explore how diverse members of the public and local leaders might view equity in terms of COVID-19 vaccine deployment.

We have begun initial discussions with community leaders and representatives to form an advisory group who will help shape the wider PCIE work, and will discuss our analysis results and help give context to their interpretation. These initial discussions will inform the scope of guided discussions with the wider panel and forum group, and will give ongoing input as the project progresses. The content of PCIE discussion groups will be shaped by the PCIE advisory group, but the example below is given to indicate the type of discussion we could focus on.

Possible discussion session: We would give a short introduction covering the vaccine prioritisation strategy, existing information about inequalities in COVID-19 mortality, and differences in population age structures. We would then guide a group discussion seeking to explore:

a. Whether the UK vaccine prioritisation plan is perceived to be equitable.
b. Public constructions of equity for vaccine prioritisation – for example what dimensions of 'need' are prioritised (exposure risk, capacity to distance/shield, mortality risk)? Is equal access for equal need appropriate, or would efforts to reduce existing health inequalities be preferable? To what extent could/should efficiency and simplicity be prioritised over short-term equity (i.e.: explore views on trade-offs between efficiency and faster overall societal gains versus maximising equity between groups).
c. Whether it would be acceptable and/or equitable to have differential vaccine priority by self-identified ethnic group or area-level income deprivation – important issues to raise here are vaccine hesitancy and perceptions of risks from vaccination in groups prioritised to receive the vaccine early after the clinical trials/approval.

We will not be able to identify health and social care staff who will also be in priority vaccination groups in the quantitative research, so will not aim to raise the equity implications of early vaccination of these workforces in the PCIE work. However, if participants raise this, we will also explore attitudes to this aspect of the vaccination strategy.

Similarly, participants may raise exceptions to the national strategy that have been reported in the media (e.g.: early prioritisation of people experiencing homelessness in Oldham), in which case perspectives will be explored.

This initial work (in particular any issues raised without prompting) will also inform the scope of a broader programme of PCIE, objective (5), and potential qualitative research by the GMPRR team in collaboration with Health Innovation Manchester.

We will ensure the proposal comes back to the RGG to check the phrasing of the results prior to any dissemination or sharing thereof.
