# Supplementary material for: Ethnic inequalities in COVID-19 vaccine uptake and comparison to seasonal influenza vaccine uptake in Greater Manchester, UK: A cohort study
Source: PLoS Med. 2022 Mar 3;19(3):e1003932. doi: 10.1371/journal.pmed.1003932 (PMC8893324; doi:10.1371/journal.pmed.1003932)
Supplement: S4 Checklist — (PDF) [file pmed.1003932.s004.pdf]

STROBE Statement—Checklist of items that should be included in reports of ***cohort studies***

	Item No	Recommendation	Section and paragraph number
**Title and abstract**	1	(*a*) Indicate the study's design with a commonly used term in the title or the abstract	Title & Abstract p2
		(*b*) Provide in the abstract an informative and balanced summary of what was done and what was found	Abstract p2-4
**Introduction**			
Background/rationale	2	Explain the scientific background and rationale for the investigation being reported	Introduction p1&2
Objectives	3	State specific objectives, including any prespecified hypotheses	Introduction p4
**Methods**			
Study design	4	Present key elements of study design early in the paper	Methods p1,2,5-9
Setting	5	Describe the setting, locations, and relevant dates, including periods of recruitment, exposure, follow-up, and data collection	Methods p1&2
Participants	6	(*a*) Give the eligibility criteria, and the sources and methods of selection of participants. Describe methods of follow-up	Methods p1 & Sup. Fig S1
		(*b*) For matched studies, give matching criteria and number of exposed and unexposed	N/A
Variables	7	Clearly define all outcomes, exposures, predictors, potential confounders, and effect modifiers. Give diagnostic criteria, if applicable	Methods p2-5
Data sources/ measurement	8*	For each variable of interest, give sources of data and details of methods of assessment (measurement). Describe comparability of assessment methods if there is more than one group	Methods p1-5
Bias	9	Describe any efforts to address potential sources of bias	Methods: Sensitivity analysis section, & Sup. Section 2
Study size	10	Explain how the study size was arrived at	Methods p1, & Sup. Fig S1
Quantitative variables	11	Explain how quantitative variables were handled in the analyses. If applicable, describe which groupings were chosen and why	Methods p2-5
Statistical methods	12	(*a*) Describe all statistical methods, including those used to control for confounding	Methods p6-10
		(*b*) Describe any methods used to examine subgroups and interactions	Methods p8
		(*c*) Explain how missing data were addressed	Methods p11 & Sup. Section 2
		(*d*) If applicable, explain how loss to follow-up was addressed	N/A
		(*e*) Describe any sensitivity analyses	Methods p11
**Results**			
Participants	13*	(a) Report numbers of individuals at each stage of study—eg numbers potentially eligible, examined for eligibility, confirmed eligible, included in the study, completing follow-up, and analysed	Results p1, & Sup. Fig S1
		(b) Give reasons for non-participation at each stage	N/A

		(c) Consider use of a flow diagram	Sup. Fig S1
Descriptive data	14*	(a) Give characteristics of study participants (eg demographic, clinical, social) and information on exposures and potential confounders	Results p2-4
		(b) Indicate number of participants with missing data for each variable of interest	Results p1 & Sup. Fig S1
		(c) Summarise follow-up time (eg, average and total amount)	Results p3
Outcome data	15*	Report numbers of outcome events or summary measures over time	Results p3 & Sup. Fig S3
Main results	16	(*a*) Give unadjusted estimates and, if applicable, confounder-adjusted estimates and their precision (eg, 95% confidence interval). Make clear which confounders were adjusted for and why they were included	Results p5-8 & Sup. Fig S4
		(*b*) Report category boundaries when continuous variables were categorized	Table 1 & Figure 1
		(*c*) If relevant, consider translating estimates of relative risk into absolute risk for a meaningful time period	N/A
Other analyses	17	Report other analyses done—eg analyses of subgroups and interactions, and sensitivity analyses	Results p5-8 & Sup. Section 2
**Discussion**			
Key results	18	Summarise key results with reference to study objectives	Discussion p1
Limitations	19	Discuss limitations of the study, taking into account sources of potential bias or imprecision. Discuss both direction and magnitude of any potential bias	Discussion p8-12
Interpretation	20	Give a cautious overall interpretation of results considering objectives, limitations, multiplicity of analyses, results from similar studies, and other relevant evidence	Discussion p13
Generalisability	21	Discuss the generalisability (external validity) of the study results	Discussion p12
**Other information**			
Funding	22	Give the source of funding and the role of the funders for the present study and, if applicable, for the original study on which the present article is based	Given in metadata with submission

*Give information separately for exposed and unexposed groups.

Note: An Explanation and Elaboration article discusses each checklist item and gives methodological background and published examples of transparent reporting. The STROBE checklist is best used in conjunction with this article (freely available on the Web sites of PLoS Medicine at http://www.plosmedicine.org/, Annals of Internal Medicine at http://www.annals.org/, and Epidemiology at http://www.epidem.com/). Information on the STROBE Initiative is available at http://www.strobe-statement.org.
