# Supplementary material for: Ethnic inequalities in COVID-19 vaccine uptake and comparison to seasonal influenza vaccine uptake in Greater Manchester, UK: A cohort study
Source: PLoS Med. 2022 Mar 3;19(3):e1003932. doi: 10.1371/journal.pmed.1003932 (PMC8893324; doi:10.1371/journal.pmed.1003932)
Supplement: Attachment — Submitted filename: Review Ethnic_Inequalities.docx [file pmed.1003932.s005.docx]

Many thanks for the opportunity to review this very interesting paper, which is of considerable public health relevance. I have some minor suggestions, which I think require clarification and which may improve the quality of this manuscript.

Abstract:

- It should be made clear that this paper is based on first/any dose of Covid-19 vaccination, and does not present information on full course of vaccination. This is clarified in the manuscript, but is relevant in the abstract too.
- It would be helpful to include any information on vaccine type(s)/make(s)

Methods:

- Was there any other form of censoring considered e.g. moving out of the region?
- Further information on full vaccination status would be helpful in the Methods, even if just to advise the reader that this was not possible to measure
- Some further elaboration on the “shielded patient list” would be helpful. This may be clear to a UK audience, but may be less clear for other international readers
- PPI section is excellent and a real strength of this paper

Results:

- The second paragraph of Descriptive Statistics section does not mention Pakistanis although they appear from the data to be the second largest ethnic group
- Table 1 under Vaccine eligibility group, are the clinical risk groups (high vs. moderate) and age groups mutually exclusive or is there overlap between some of these? Can this be clarified? If I understand correctly, you assigned individuals to whichever eligibility group they first fell in to, but what did this mean in practice for most older patients? Were they more likely to be in the moderate or high clinical risk groups (based on comorbidities), or more likely to be in their respective age groups? A footnote in this table may be helpful.
- Summary data are presented in Table 1 for death during follow-up. Is there any information on what proportion of deaths might have been attributed to Covid (either as primary or contributory cause)? Moreover, any summary data on Covid infection rates may be helpful for context if available.
- The findings relating to inequalities in vaccine uptake appear to be quite robust and are important to note. It would be helpful to contextualise this further by translating some of the key results (which are all presented in relative terms using HRs etc) in to absolute numbers if possible. For this population of approx. 1.1 million people, how do these results translate in to numbers of missed vaccines for certain ethnicities or certain risk groups? Translation in to absolute numbers may be particularly helpful for public health messaging.
- Did you have any information on length of time residing in the UK? These individuals are all registered with GPs but I do not get a sense whether they are all long-standing UK citizens who are otherwise integrated in to UK society, or if they might be relatively recent arrivals and still heavily influenced by cultural/health beliefs in their countries of origin. Or perhaps it is the case that different age groups have differentially integrated to UK society. Length of time residing in UK may be an important confounder, particularly considering the more marked inequalities observed among older age groups. Inequalities appear to be widest in those who are older in age or extremely clinically vulnerable (and as per above query – perhaps those who are more clinically vulnerable are also by default older in age because of increased risk of comorbidities?). One would also expect that the length of time individuals have been living in UK will affect/influence their acceptance of UK vaccine-related messaging, as well as likelihood of language barriers etc?
- Following on from this, did you have any information on whether some participants were asylum seekers or recently-arrived refugees? I’m sure this would only be a small minority (and likely to be very ethnically diverse, so may not allow the same level of detailed analysis), but they are a particularly important subgroup nonetheless.
- The results relating to the Bangladeshi population are interesting and warrant some further explanation or clarification. From Figure 1 C it appears that they had higher uptake of influenza vaccine vs. White British population in 2019/20. Figure 2B suggested that this was particularly the case in more deprived areas. This alone is an interesting finding. But Table S1 suggests that influenza vaccine uptake was lower in the Bangladeshi population vs. White British population in 2019/20. If I understand correctly, this is because of differences in eligibility groups between Bangladeshi and GM population (per Table S3). It would be very useful to acknowledge this finding in the manuscript as it is not entirely intuitive.
