# Supplementary material for: Ethnic inequalities in COVID-19 vaccine uptake and comparison to seasonal influenza vaccine uptake in Greater Manchester, UK: A cohort study
Source: PLoS Med. 2022 Mar 3;19(3):e1003932. doi: 10.1371/journal.pmed.1003932 (PMC8893324; doi:10.1371/journal.pmed.1003932)
Supplement: Attachment — Submitted filename: Response_to_reviewers.docx [file pmed.1003932.s006.docx]

We would like to thank each of the reviewers and editors for their very constructive and thoughtful comments. We have now fully revised our manuscript, taking into account all of the comments, and believe this has substantially improved the manuscript. We have added detail and important contextual information to each of the manuscript sections, added an additional analysis stratifying by gender, improved the clarity of several figures, and have acknowledged additional limitations of our analytical approach. Specifically, we have addressed each comment as indicated in our point-by-point response below.

**Comments from the academic editor:**

There should be some more information about how persons, who are eligible for a vaccination, get that information. Do they receive an invitation letter? And is this letter in different languages? Are there any additional efforts to reach and motivate people to make a vaccination appointment? Especially for non-UK readers, it is important to understand how the system works.

Thank you for this comment, we have now provided additional information in the introduction about the ways in which people were informed about vaccination eligibility and how they were able to book.

L88: *“Access to Covid-19 vaccination initially required an appointment booked in advance via the ‘national booking service’, predominantly an online booking service available only in English, and which required users to be registered with a General Practice (GP) surgery[8]. Many people booked appointments following public announcements indicating that their group was eligible to book, and some individuals received letters in the post and/or text messages or telephone calls from their GP inviting them to book an appointment through the national booking service. During the first few months of the Covid-19 vaccine rollout, the majority of vaccination appointments available were at out-of-town mass vaccination centres or hospital hubs. However, as the rollout has progressed, vaccinations have increasingly been provided without the need for pre-booked appointments and at local and pop-up sites, and information is increasingly (though not consistently) available in additional non-English languages.”*

**Comments from the reviewers:**

**Reviewer #1**: This study reveals the vaccine uptake inequalities by using a reliable public dataset. The results, including sensitivity analyses and public discussion, seem to justify the authors' conclusions. Please consider the following minor comments.

#1 L35

Adding more detail about the pandemic situation in England will help me understand. How much is the COVID-19 morbidity or mortality among the older adults from minority ethnic groups compared to White British adults in England?

We are grateful for this comment and have added the following text to the introduction:

L73: *“Older adults from minority ethnic groups have been particularly at risk, with Covid-19 mortality rates in England more than 3-fold higher amongst people belonging to Black ethnic groups, and more than 2-fold higher amongst those from Asian ethnic groups, compared to rates amongst those from White ethnic groups[2]. These disproportionate impacts are likely due in part to socioeconomic disadvantages and wide existing health inequalities amongst those belonging to minority ethnic groups compared to White British adults in England[2,3]”*

#2 L43, L338

How are booking or travelling to vaccination appointments difficult for ethnic minority groups in England? Is it available through the internet, telephone, or hand-writing? How far are the vaccination centres are from their residence? Is there no language support? Are the workers available to access vaccination at night or their workplace?

We agree this is information adds vital context, and have added the following section to the introduction:

L88: *“Access to Covid-19 vaccination initially required an appointment booked in advance via the ‘national booking service’, predominantly an online booking service available only in English, and which required users to be registered with a General Practice (GP) surgery[8]. Many people booked appointments following public announcements indicating that their group was eligible to book, and some individuals received letters in the post and/or text messages or telephone calls from their GP inviting them to book an appointment through the national booking service. During the first few months of the Covid-19 vaccine rollout, the majority of vaccination appointments available were at out-of-town mass vaccination centres or hospital hubs. However, as the rollout has progressed, vaccinations have increasingly been provided without the need for pre-booked appointments and at local and pop-up sites, and information is increasingly (though not consistently) available in additional non-English languages.”*

#3 L329

I suppose fewer opportunities existed to reflect ethnic minority groups' voices in the new-introduced vaccination system. The rapid political decision-making process might play a significant role in such an unusual inequality.

We would like to thank the reviewer for raising this important point. We have added the following to the discussion:

L387: *“It is unclear whether any type of health equity impact assessment or community engagement was undertaken during the very rapid centralised design of the Covid-19 vaccine rollout, and this may have contributed to the observed exacerbation of inequalities.”*

#4 L351

I think the other sociodemographic factors might be the confounders in this study. The Covid-19 vaccine uptake may also differ across the participants' years of residence, occupation, or having family members.

We agree with the reviewer that other sociodemographic factors are highly likely to influence vaccine uptake. However, by focusing on the comparison between Covid-19 vaccine uptake inequalities and Influenza vaccine inequalities for the same group of individuals, we demonstrate that when all other sociodemographic factors are unchanged there were markedly different outcomes in vaccine uptake inequalities between the two vaccines. In order to highlight the role of other sociodemographic factors we have added the following to the discussion:

L420: *“Analysis stratified by gender indicated ethnic inequalities in vaccine uptake were moderately wider for women compared to men for many minority ethnic groups. This highlights the interactions between sociodemographic factors that may exacerbate health inequalities amongst some groups. However, we were unable to explore the role of other factors that may intersect with ethnicity and affect vaccination uptake, such as faith, country of birth, or preferred spoken and written languages, as these are rarely recorded in electronic health records.”*

Is there any possibility the participants had already got vaccinated in the other area? Does the database cover the population only who has permanent citizenship?

We are grateful for the opportunity to clarify these details, and have added the following to the methods section:

L129: *“Vaccinations appear in GM residents’ GMCR record whether they received vaccination within or outside of GM”*

L117: *“…electronic health record data on approximately 2.8 million patients registered with GP surgeries in Greater Manchester (GM), independently of residence or citizenship status[15]”*

**Reviewer #2:** The authors present a detailed report of COVID-19 vaccination uptake among different racial/ethnic groups from a large multicultural city in the United Kingdom. Using survival analysis in an enormous data set, they give compelling evidence of disparities in vaccination uptake between groups. They additionally show how these disparities persist when stratified by deprivation, age category and vaccine eligibility group. Throughout, they contextualise COVID-19 vaccination uptake by comparing it to influenza vaccination uptake prior to the start of the pandemic. All in all, these results suggest that existing racial/ethnic disparities in vaccination uptake were exacerbated by the pandemic. This is vital evidence to encourage health policy makers to act to address these disparities and, additionally, for health researchers to investigate the specific causes of these disparities.

I thought this was a very well-written manuscript and I appreciated the great level of detail the authors went to to present their findings, including a rigorous exploration of bias due to missing data and sensitivity analyses. A few minor questions to address follow:

Introduction

-The final sentence of the penultimate and ultimate paragraphs (i.e. the sentences starting "However, these studies have focused..." and, "We use this comparison because similar groups...") could be rephrased to make it clearer that your study is using the influenza vaccine uptake comparison as an example of a pre-pandemic disparity in routine vaccine uptake. I suggest rephrasing the first sentence I referenced like so: "..., and have not been compared to inequalities in uptake of other vaccination programmes." The final sentence in the Introduction could be rephrased to: "...this allows us to see if the inequalities in vaccination uptake existed before." As it stands, these sections make me think you want to show that, if your study had revealed no disparity in influenza vaccination uptake by racial/ethnic group, that would be evidence that there were no such health disparities at all (i.e. in vaccination programme uptake or beyond).

We really appreciate this suggested rephrasing and have updated those sentences as suggested:

L99: *“However, these studies have focused on specific population groups, lacked granularity in ethnic group definitions, and have not been compared to inequalities in uptake of other vaccination programmes.”*

L108: *“…seasonal Influenza vaccination[16] and so this allows us to see if the inequalities in vaccination uptake existed before.”*

Methods

-Data Collection: Please report why data were unavailable for one surgery, and where it was located

We apologise for the inaccuracy here – when checking where the surgery was located we realised there are actually 2 surgeries for which data were unavailable. We have now updated the methods sections and added the further information as requested:

L118: *“This gives almost population-level coverage, with data from 433 of 435 (99.5%) GP surgeries in the region. The two GP surgeries that do not contribute data have chosen to opt out of data sharing into the GMCR. For reference, one is located in Tameside and the other is in Bolton.”*

-Exposures: Although the number of participants in the Gypsy or Irish Traveller group may be too small to report meaningful statistics in this study, perhaps reporting them in the supplementary materials would be helpful for potential future researchers wishing to explore vaccination patterns in this ethnic group via meta-analysis.

We appreciate this suggestion and agree it could be helpful for further research into this underserved group, but are not able to report these statistics as the very low numbers would breach the disclosure rules under which we obtained access to this dataset. However, we would like to acknowledge this more clearly as a limitation, so have added the following to the discussion:

L415: *“However even with this large sample, we were unable to estimate inequalities in vaccine uptake for the Gypsy or Irish Traveller group due to very low numbers.”*

-Statistical analyses: The Efron method for handling ties is generally less biased than the Breslow method (see Hertz-Picciotto and Rockhill 1997: Validity and Efficiency of Approximation Methods for Tied Survival Times in Cox Regression). Why did you choose the Breslow method instead?

We would like to thank the reviewer for this comment and have added the following text to the methods and discussion sections to explain that we used the less computationally intensive Breslow method for pragmatic reasons. Importantly, given our very large sample size, we do not think this is likely to have introduced substantial bias. However, we have added this as a limitation of the analysis, and cited the paper referenced by the reviewer (new ref 32). We attempted to run a sensitivity analysis using the Efron method, but were unable to get this model to run with the computer available to us in the secure research environment.

Methods L197: *“… the Breslow method for handling ties. We used the Breslow method for pragmatic reasons as this is much less computationally intensive than other methods, particularly for a large dataset.”*

Discussion L436: *“Another limitation of our analysis is that we used the Breslow method to handle ties in the Cox regression models, as opposed to the Efron method which has been shown to generally be less biased[32]. Although this may have biased our results to underestimate inequalities in vaccine uptake, this method is more computationally feasible with a large dataset and is unlikely to have markedly biased estimates given our large sample size[32].”*

Results

-Ethnic inequalities in Covid-19 vaccine uptake: The way the hazard ratio ranges are reported in the text is rather difficult to read; it can be hard to disentangle the 95% confidence interval values from the point estimates. I suggest either omitting the 95% confidence interval values from the text (because they are reported in Table S2 and quite narrow anyway) or changing the format to something like: "Hazard ratio (HR) range 0.276 (95% CI: 0.241, 0.317) to 0.471 (95% CI: 0.450, 0.494)."

We are grateful for this suggestion which we agree improves the readability of the results section. We have also reduced the number of decimal places reported in the main text at the suggestion of another reviewer. We have updated throughout, with an example given below.

L279: *“Compared to the White British group, uptake differences tended to be largest for Black African (Hazard ratio (HR) range: 0.28 (95%ci 0.24 to 0.32) to 0.47 (95%ci 0.45 to 0.49)), Black Caribbean (HR range 0.36 (95%ci 0.33 to 0.38) to 0.57 (95%ci 0.51 to 0.64)) ‘other Black background’ (HR range 0.38 (95%ci 0.29 to 0.50) to 0.48 (95%ci 0.40 to 0.57)) and Arab groups (HR range 0.38 (95%ci 0.30 to 0.49) to 0.54 (95%ci 0.40 to 0.74)).”*

-I am curious to see how these results differed by gender within racial/ethnic groups. Could you please report a subgroup analysis (e.g. in the same format as Figure 2) showing vaccine uptake patterns by racial/ethnic group stratified by gender?

We appreciate this interesting suggestion, and have now included the suggested analysis along with the following additions to the manuscript. In brief, we found that Covid-19 vaccine uptake was slightly higher amongst women compared to men, but that inequalities in vaccine uptake between ethnic groups were moderately wider for women compared to men. Overall, inequalities in both Covid-19 and Influenza vaccine uptake followed similar patterns amongst women and men.

Methods:

L199: *“We also estimated associations between ethnicity and time-to-vaccine uptake adjusted by vaccine eligibility group (age and clinical risk groups) amongst those eligible for both Covid-19 vaccination and 2019/20 seasonal Influenza vaccination. We repeated adjusted analysis for both Covid-19 and Influenza vaccine uptake amongst this population stratified by gender.”*

Figures and tables:

Table S1 (unadjusted vaccine uptake): rows added to show vaccine uptake amongst men vs women

New Figure S4: Associations between ethnic group and vaccine uptake by gender

New Table S6: Associations between ethnic group and vaccine uptake by gender

Figure S4:

Results:

L268: *“Covid-19 vaccine uptake was slightly higher amongst women (83.24% (95%ci 83.15 to 83.33)) than men (81.70% (95%ci 81.60 to 81.80%)).”*

L245: *“Influenza vaccine uptake was slightly lower amongst women (52.93% (95%ci 52.78 to 53.08)) compared to men (53.78% (95%ci 53.62 to 53.94)).”*

L274: *“Inequalities in vaccine uptake between ethnic groups followed very consistent trends amongst men as compared to women, though the magnitudes of inequalities were moderately greater for women than men for many minority ethnic groups (10 of 16 for Covid-19 vaccination, 7 of 16 for Influenza vaccine) (Figure S4 & Table S6). There were no minority ethnic groups for whom inequalities in vaccine uptake were statistically significantly greater amongst men than women.”*

Discussion:

L420: *“Analysis stratified by gender indicated ethnic inequalities in vaccine uptake were moderately wider for women compared to men for many minority ethnic groups. This highlights the interactions between sociodemographic factors that may exacerbate health inequalities amongst some groups.”*

**Reviewer #3: Alex McConnachie, Statistical Review**

The paper by Watkinson et al examines variations in COVID-19 vaccination uptake in 2020-21 between ethnic groups, and compares these with variations in flu vaccination uptake in 2019-20. This review considers the use of statistics in the paper.

Overall, the statistical methods and presentation is very good. The main analyses are supported by a range of adjusted, stratified, and sensitivity analyses, and the results are not over-interpreted. My comments are quite minor.

For my taste, there are too many decimal places reported. I think one less could be used throughout the paper.

We have edited all figures reported in the main text to have 2 decimal places

Figure 3 might work better with the two panels overlaid on the same plot.

We agree with the reviewer that overlaying the panels may help with visual comparison of the two sets of results. However, throughout the manuscript we have been careful to only overlay results that correspond to the exact same population of individuals, such that results are directly comparable. Since these plots show results from distinct subpopulations we have decided to keep them on separate axes to maintain this distinction.

The Kaplan-Meier plots show survival probabilities, which is not incorrect, but may be confusing to a general readership, since the paper is not about mortality. The majority of the paper talks in terms of vaccine uptake. Would it therefore be better to flip the y-axis of the K-M plots, and show the cumulative probability of vaccination?

We are grateful for this suggestion and agree that flipping the plots gives are more intuitive figure. We have replotted these results and have updated Figure S3. We have also edited the methods and figure legend to reflect this change:

L186: *“We estimated the cumulative probability of vaccination over time stratified by Covid-19 vaccination eligibility group for both Covid-19 vaccination (first-dose) and seasonal Influenza vaccination, and presented results as Kaplan-Meier failure curves with 95% confidence intervals.”*

Figure S3 legend: *“Kaplan-Meier failure curves indicating the cumulative probability of vaccination over time for (A) Covid-19 and (B) Influenza vaccines, stratified by Covid-19 vaccination eligibility group.”*

Looking at Figure S3, I assume the order of the legends is in terms of the final vaccination probability? Perhaps this would be clearer if the probabilities were shown (with 95% CIs?) as part of the legend.

We appreciate the opportunity to clarify the ordering in Figure S3. The legend order shows the order in which groups became eligible for vaccination. We have amended the figure legend to clarify this.

Figure S3 legend: *“…Individuals were assigned to the highest priority vaccine eligibility group for which they were eligible. Priority proceeded from those aged 80+ down the age brackets, with the high clinical risk group eligible at the same time as the 70-74 age group, and the moderate clinical risk group eligible between the 65-69 and 60-64 age groups, as indicated in the legend group order. Vaccine eligibility groups are mutually exclusive, such that (for example) an individual aged 80 with high clinical vulnerability would be in the 80+ age group, whereas an individual aged 65 with high clinical vulnerability would be in the high clinical vulnerability group.”*

Also, I'm not sure if it is my eyesight, but the colours in the legend do not seem to match the colours of the curves.

We are grateful to the reviewer for noticing this inconsistency. The transparency was set to the wrong level, which made the colours in the legend slightly lighter than those in the figure. We have now amended this so that the colours match (see above).

Figure S3 is quite cramped, with 3 estimates and CIs per ethnic group. Could the y axis be stretched a little?

We think the reviewer may be referring to the original Figure S4, which shows 3 estimates with different adjustments: “Associations between ethnic group and Covid-19 vaccine uptake – sensitivity analysis adjusting by locality or income deprivation”. We agree that the figure looked cramped, and have elongated the y axis as suggested, as well as making the symbol size smaller to help with visual clarity. This is presented as updated Figure S5.

In Figure S6, a slight vertical offset to separate each pair of estimates and CIs, as in other similar figures, would help.

We are grateful for this suggestion and have updated the Figure (now Figure S7) to include the offset.

The sensitivity analysis that includes geographic adjustment uses 10 LA areas, which is fine. Not for this paper, but would it be possible to do (and worth doing) an analysis by LSOA, adjusting for spatial correlation, and explore the associations between uptake and factors such as distance to nearest vaccination centre?

We agree this is a really interesting idea for future research, and is something we’re hoping to look at. Unfortunately, we do not currently have full information about when and where smaller or pop-up vaccination centres were in operation, and do not have access to information about where vaccines were received. It would therefore be difficult to reliably estimate distance to vaccination centre, but we hope this may be possible in future. Another consideration is whether we would be able to capture ‘functional’ or ‘travel time’ distance to vaccine centres, as this likely highly dependent on whether individuals have access to private cars or are using public transport.

**Reviewer #4**: Many thanks for the opportunity to review this very interesting paper, which is of considerable public health relevance. I have some minor suggestions, which I think require clarification and which may improve the quality of this manuscript.

Abstract:

-	It should be made clear that this paper is based on first/any dose of Covid-19 vaccination, and does not present information on full course of vaccination. This is clarified in the manuscript, but is relevant in the abstract too.

We agree that this is important to clarify and have added the following edits to the abstract make this more explicit and acknowledge it as a limitation.

L14: *“… and analysed first-dose vaccination only”*

L32: *“Another limitation of our approach is the focus only on uptake of first doses of Covid-19 vaccination.”*

-	It would be helpful to include any information on vaccine type(s)/make(s)

We appreciate this suggestion, and have clarified in the abstract and methods that we included vaccination with any approved Covid-19 vaccine.

Abstract L14: *“We included vaccination with any approved Covid-19 vaccine”*

Methods L127: *“We included data on date of first-dose Covid-19 vaccination (all approved Covid-19 vaccine types)”*

Methods:

-	Was there any other form of censoring considered e.g. moving out of the region?

We are grateful for this important point. We did not have reliable data on any other form of censoring, and have added a statement to the methods section to indicate this.

L194: *“No other form of censoring was included. It is possible a small proportion of individuals moved away from the area during the 138-day analysis period but this is not reliably recorded in the GMCR.”*

However, if individuals received vaccination outside of the area this would still appear in their health record, and we have now added text to the methods section to clarify this.

L129: *“Vaccinations appear in GM residents’ GMCR record whether they received vaccination within or outside of GM”*

-	Further information on full vaccination status would be helpful in the Methods, even if just to advise the reader that this was not possible to measure

We have added “first-dose” to the statement in the variables section of the methods (below). As indicated in response to the comment above, we have also clarified in the abstract that we analyse first-dose of Covid-19 vaccination only (L14), and acknowledge this as a limitation both in the abstract (L32) and discussion (L441). We have also clarified that a single dose of the seasonal Influenza vaccine is full vaccination.

L127: *“We included data on date of first-dose Covid-19 vaccination”*

L129: *“(the seasonal influenza vaccine is a single dose only vaccine)”*

-	Some further elaboration on the "shielded patient list" would be helpful. This may be clear to a UK audience, but may be less clear for other international readers

We agree with the reviewer that more information is needed here, especially for an international audience. We have added the following information and an additional reference to the methods section:

L137: *“… the ‘Shielded Patient List’ who were eligible for Covid-19 vaccination in priority group 4[5]. The clinical risk criteria for the Shielded Patient List was published by NHS Digital and included patients with severe respiratory conditions such as cystic fibrosis and severe chronic obstructive pulmonary disease, those with immunosuppression due to health conditions or treatments, and patients undergoing kidney dialysis[17].”*

Reference 17: NHS Digital. Shielded patient list risk criteria. 2020 [cited 12 Dec 2021]. Available: https://digital.nhs.uk/coronavirus/shielded-patient-list/risk-criteria

-	PPI section is excellent and a real strength of this paper

Thank you for this comment. We really appreciate this acknowledgement of the major contribution our public contributors made in helping shape this work.

Results:

-	The second paragraph of Descriptive Statistics section does not mention Pakistanis although they appear from the data to be the second largest ethnic group

We are very grateful to the reviewer for bringing this omission to our attention. We have now updated the text as follows:

L258: *“The largest minority ethnic groups were Pakistani (50,268 – 4.57%), ‘any other White background’ (43,195 – 3.93%), …”*

-	Table 1 under Vaccine eligibility group, are the clinical risk groups (high vs. moderate) and age groups mutually exclusive or is there overlap between some of these? Can this be clarified? If I understand correctly, you assigned individuals to whichever eligibility group they first fell in to, but what did this mean in practice for most older patients? Were they more likely to be in the moderate or high clinical risk groups (based on comorbidities), or more likely to be in their respective age groups? A footnote in this table may be helpful.

We appreciate the opportunity to clarify the vaccine eligibility groupings used. We have added the following footnote to Table 1, and have also added the same information to the figure legend of Figure S3.

Table S1 footnote: *“^3^ Individuals were assigned to the highest priority vaccine eligibility group for which they were eligible. Priority proceeded from those aged 80+ down the age brackets, with the high clinical risk group eligible at the same time as the 70-74 age group, and the moderate clinical risk group eligible between the 65-69 and 60-64 age groups. Vaccine eligibility groups are mutually exclusive, such that (for example) an individual aged 80 with high clinical vulnerability would be in the 80+ age group, whereas an individual aged 65 with high clinical vulnerability would be in the high clinical vulnerability group.”*

-	Summary data are presented in Table 1 for death during follow-up. Is there any information on what proportion of deaths might have been attributed to Covid (either as primary or contributory cause)? Moreover, any summary data on Covid infection rates may be helpful for context if available.

We agree with the reviewer that this information could add very useful context to our results. However, we do not have reliable data on Covid-19 infection rates from our dataset, as community testing data is not currently included in data feeds to the GMCR. Similarly, we are unable to reliably attribute Covid-19 as a cause of death from our dataset, though work to achieve this is underway.

-	The findings relating to inequalities in vaccine uptake appear to be quite robust and are important to note. It would be helpful to contextualise this further by translating some of the key results (which are all presented in relative terms using HRs etc) in to absolute numbers if possible. For this population of approx. 1.1 million people, how do these results translate in to numbers of missed vaccines for certain ethnicities or certain risk groups? Translation in to absolute numbers may be particularly helpful for public health messaging.

We agree with the reviewer that absolute differences in vaccine uptake can also be important for contextualising the magnitude of differences, and for public health messaging and planning. However, absolute numbers can also be counterintuitive when compared to relative inequalities, given the much smaller population sizes and typically younger age structures of minority ethnic groups. We have provided percentage vaccine uptake at the end of the analysis period standardised by vaccine eligibility groups (Table S3), which gives a more absolute difference in uptake, and will allow readers to calculate additional metrics such as differences per million population with the demographic information provided. To emphasise these statistics, we have added a statement about inequalities in standardised Influenza vaccine uptake to the main text in the results section to mirror the statement about differences in percentage uptake of the Covid-19 vaccine.

L304: *“Similarly, Influenza vaccine uptake at the end of the analysis period (standardised by vaccine eligibility group) was over three percentage points higher for the Bangladeshi and ‘other ethnic group’ groups compared to the White British group (Table S3).”*

L287: *“In line with these results, there were substantial differences in standardised total vaccine uptake at the end of the analysis period, with eligibility group-standardised uptake at least 20 percentage points lower than the White British group for 6 of 16 minority ethnic groups (Table S3).”*

-	Did you have any information on length of time residing in the UK? These individuals are all registered with GPs but I do not get a sense whether they are all long-standing UK citizens who are otherwise integrated in to UK society, or if they might be relatively recent arrivals and still heavily influenced by cultural/health beliefs in their countries of origin. Or perhaps it is the case that different age groups have differentially integrated to UK society. Length of time residing in UK may be an important confounder, particularly considering the more marked inequalities observed among older age groups. Inequalities appear to be widest in those who are older in age or extremely clinically vulnerable (and as per above query - perhaps those who are more clinically vulnerable are also by default older in age because of increased risk of comorbidities?). One would also expect that the length of time individuals have been living in UK will affect/influence their acceptance of UK vaccine-related messaging, as well as likelihood of language barriers etc?

Following on from this, did you have any information on whether some participants were asylum seekers or recently-arrived refugees? I'm sure this would only be a small minority (and likely to be very ethnically diverse, so may not allow the same level of detailed analysis), but they are a particularly important subgroup nonetheless.

We are grateful to the reviewer for raising these important points. We have added the following to the discussion section to acknowledge the likely complex influence of these factors, and to explain that we were unable to investigate these directly because we don’t have that data.

L421: *“This highlights the interactions between sociodemographic factors that may exacerbate health inequalities amongst some groups. However, we were unable to explore the role of other factors that may intersect with ethnicity and affect vaccination uptake, such as faith, country of birth, or preferred spoken and written languages, as these are rarely recorded in electronic health records.”*

-	The results relating to the Bangladeshi population are interesting and warrant some further explanation or clarification. From Figure 1 C it appears that they had higher uptake of influenza vaccine vs. White British population in 2019/20. Figure 2B suggested that this was particularly the case in more deprived areas. This alone is an interesting finding. But Table S1 suggests that influenza vaccine uptake was lower in the Bangladeshi population vs. White British population in 2019/20. If I understand correctly, this is because of differences in eligibility groups between Bangladeshi and GM population (per Table S3). It would be very useful to acknowledge this finding in the manuscript as it is not entirely intuitive.

We would like to thank the reviewer for raising this point. The reviewer is correct that the differences between percent uptake and standardised percent uptake are driven by the much younger age structure of most minority ethnic groups (including the Bangladeshi group) relative to the White British group in England. We have added a footnote to table S1, which shows unadjusted percent vaccine uptake, to acknowledge this to help readers make sense of the differences in results between Tables S1 and S3.

Table S1 footnote: *“^2^ More income-deprived quintiles and most minority ethnic groups have younger population age structures relative to less income-deprived groups and the White British group respectively, so % uptake figures presented in this table should be interpreted with caution as trends are substantially confounded by age”*

We also agree that the relatively higher eligibility group-standardised Influenza vaccine uptake amongst some minority ethnic groups compared to the White British group is worth noting more explicitly in the main text, so have added the following to the results section:

L304: *“Similarly, Influenza vaccine uptake at the end of the analysis period (standardised by vaccine eligibility group) was over three percentage points higher for the Bangladeshi and ‘other ethnic group’ groups compared to the White British group (Table S3).”*

**Requests from the editors:**

Title: Please update title to ‘Ethnic inequalities in Covid-19 vaccine update and comparison to seasonal Influenza vaccine update in Greater Manchester: a cohort study’.

We have updated the title as requested

Financial disclosure: Please provide more information (see here for details of what is required): <https://journals.plos.org/plosmedicine/s/submission-guidelines#loc-financial-disclosure-statement>

Abstract Methods and Findings:

* Please include the study design, time period during which the study took place, length of follow up, and main outcome measures.

We have added the following details to the abstract:

L10: *“We conducted a retrospective cohort study”*

L13: *“between 1st December 2020 and 18th April 2021”*

L14: *“(138 days follow-up)”*

Main outcome measures, L12: *“time-to-Covid-19 vaccination” & L14/15: “vaccination with any approved Covid-19 vaccine, and analysed first-dose vaccination only”*

* Please include basic demographic data (summarising that presented on lines 198 – 201).

We have added the following demographic information:

L19: *“The majority of individuals (871,231 – 79.24%) were White British. The largest minority ethnic groups were Pakistani (50,268 – 4.75%), ‘any other White background’ (43,195 – 3.93%), ‘any other ethnic group’ (34,568 – 3.14%) and Black African (18,802 – 1.71%).”*

* Please include the important dependent variables that are adjusted for in the analyses.

Added L16: *“adjusting by age group and clinical risk”*

* In the last sentence of the Abstract Methods and Findings section, please describe the main limitation(s) of the study's methodology.

We have added the following:

L30: *“One limitation of this study stems from the incomplete granularity of ethnicity recording in electronic health records, meaning that some of the largest minority ethnic groupings were ‘any other’ categories. There is likely to be substantial diversity within these groupings, which may have masked health inequalities. Another limitation of our approach is the focus only on uptake of first doses of Covid-19 vaccination.”*

Line 27-8: Please update to ‘This suggests that the Covid-19 vaccination programme has created…’.

We have updated this.

L37: *“This suggests the Covid-19 vaccination programme has created additional…”*

At this stage, we ask that you include a short, non-technical Author Summary of your research to make findings accessible to a wide audience that includes both scientists and non-scientists. The Author Summary should immediately follow the Abstract in your revised manuscript. This text is subject to editorial change and should be distinct from the scientific abstract. Please see our author guidelines for more information: <https://journals.plos.org/plosmedicine/s/revising-your-manuscript#loc-author-summary>

We have added an Author Summary as below.

L42: **Author summary**

**Why was this study done?**

- Previous research has found wide disparities in early Covid-19 vaccine uptake between ethnic groups in many countries
- Uptake of Covid-19 vaccination was particularly low amongst older adults belonging to Black or Black British ethnic groups in the UK
- However, previous research has tended to use broad ethnic groupings, and ethnic inequalities in Covid-19 vaccination uptake have not been contextualised by comparison with inequalities in uptake of previous vaccination programmes

**What did the researchers do and find?**

- We used electronic health records for the population of Greater Manchester, England to estimate inequalities in Covid-19 and seasonal Influenza vaccine uptake between 17 ethnic groups
- We found that ethnic inequalities in Covid-19 vaccine uptake are far wider than those seen previously for seasonal Influenza vaccine uptake, and exist even amongst those recently vaccinated against Influenza
- Ethnic inequalities in Covid-19 vaccine uptake are concentrated amongst the most vulnerable – those living in the most deprived neighbourhoods, and older and extremely clinically vulnerable adults

**What do these findings mean?**

- These findings suggest the Covid-19 vaccine rollout has exacerbated pre-existing health inequalities in vaccine uptake
- Themes raised in our public and community discussion groups suggest both lower trust in Covid-19 vaccines and practical barriers to vaccine access likely contribute to lower Covid-19 vaccine uptake amongst minority ethnic groups
- Further research and community engagement is needed to build trust and confidence amongst minority ethnic communities, and to better understand and remove barriers to vaccine access

Citations should be in square brackets, and preceding punctuation.

We have edited this throughout

Please remove the ‘Role of funders statement’ (Line 175) and instead update your Financial disclosure (submission form).

We have removed this section and completed the financial disclosure form

Please remove the ‘Competing interests statement’ (Line 188) and instead include this information in your submission form. In the event of publication, this information is included as metadata.

We have removed this section and included the information in the submission form

Please ensure that the study is reported according to the STROBE guideline, and include the completed STROBE checklist as Supporting Information. Please add the following statement, or similar, to the Methods: "This study is reported as per the Strengthening the Reporting of Observational Studies in Epidemiology (STROBE) guideline (S1 Checklist)."

The STROBE guideline can be found here: <http://www.equator-network.org/reporting-guidelines/strobe/>

When completing the checklist, please use section and paragraph numbers, rather than page numbers.

We have updated the checklist to use section and paragraph numbers, and uploaded the checklist as a supplementary information file. We have added the reporting statement to the methods section.

L250: *"This study is reported as per the Strengthening the Reporting of Observational Studies in Epidemiology (STROBE) guideline (S1 Checklist)."*

Did your study have a prospective protocol or analysis plan? Please state this (either way) early in the Methods section.

a) If a prospective analysis plan (from your funding proposal, IRB or other ethics committee submission, study protocol, or other planning document written before analyzing the data) was used in designing the study, please include the relevant prospectively written document with your revised manuscript as a Supporting Information file to be published alongside your study, and cite it in the Methods section. A legend for this file should be included at the end of your manuscript.

b) If no such document exists, please make sure that the Methods section transparently describes when analyses were planned, and when/why any data-driven changes to analyses took place.

c) In either case, changes in the analysis-- including those made in response to peer review comments-- should be identified as such in the Methods section of the paper, with rationale.

We have added an appendix with our prospective analysis plan along with information about deviations from the original plan and reference these in the methods section.

Added to methods L177: *“We followed a prospective analysis plan which was submitted with the application to use the GMCR data. This is provided as a Supplementary Information file, along with an explanation of the changes we made. The rationale for the main changes is outlined in the public and community involvement and engagement section below.”*

Please remove subheadings from your Discussion.

We have removed the subheadings

Please re-order your discussion such that comparison to the literature and interpretation precedes strengths and limitations.

We have reordered the discussion as requested

Please remove funding details from your acknowledgements and include them in the Financial disclosure (submission form). Specific grant numbers should be included.

We have removed the funding details and included them in the financial disclosure form

Please remove the ‘Author contributions’ section (similar to comments above, this information will be included as metadata based on submission form responses in the event of acceptance).

We have removed this section

When citing preprints (e.g. reference 13), please include [preprint] per the instructions here: <https://journals.plos.org/plosmedicine/s/submission-guidelines#loc-references>

We have updated the preprint reference as requested

Please define the abbreviations in Figure S1.

We have defined the abbreviations and added the following text to the Figure S1 legend:

*“Abbreviations: Greater Manchester (GM), General Practice (GP), Lower-layer Super Output Area (LSOA) (LSOAs are neighbourhood-level administrative boundaries containing approx. 1000 residents).”*
