# Supplementary material for: Ethnic inequalities in COVID-19 vaccine uptake and comparison to seasonal influenza vaccine uptake in Greater Manchester, UK: A cohort study
Source: PLoS Med. 2022 Mar 3;19(3):e1003932. doi: 10.1371/journal.pmed.1003932 (PMC8893324; doi:10.1371/journal.pmed.1003932)
Supplement: Attachment — Submitted filename: Response_to_reviewers.docx [file pmed.1003932.s007.docx]

**Requests from Editors:**

Please update your title to 'Ethnic inequalities in Covid-19 vaccine uptake and comparison to seasonal Influenza vaccine uptake in Greater Manchester, UK: a cohort study.

We have updated the title (page 1)

The Data Availability Statement (DAS) requires revision. I feel the URL included provides detailed information for those who have provided their data for research purposes but does not provide sufficient information for researchers wishing to access the data – please provide an additional URL/email address suitable for researcher enquiries.

We have added a contact email address for researcher enquiries:

… *For further details please see* [*https://healthinnovationmanchester.com/thegmcarerecord/the-gm-care-record-forsecondary-uses-research/*](https://healthinnovationmanchester.com/thegmcarerecord/the-gm-care-record-forsecondary-uses-research/) *or contact* [*GMCR-ops@manchester.ac.uk*](mailto:GMCR-ops@manchester.ac.uk)*.*

Please consider quantifying key findings from the ethnic inequalities in influenza vaccine update in your abstract (e.g. those found on lines 301-2).

We have added the following summary of inequalities in influenza vaccine uptake to the abstract:

Line 25: *55.71% (419,314/752,715) of eligible individuals took up Influenza vaccination. Compared to the White British group, inequalities in Influenza vaccine uptake were widest amongst White and Black Caribbean (HR 0.63 95%ci 0.58 to 0.68) and White and Black African (HR 0.67 95%ci 0.63 to 0.72) groups. In contrast, uptake was slightly higher than White British amongst ‘other ethnic group’ (HR 1.11 95%ci 1.09 to 1.12) and Bangladeshi (HR 1.08 95%ci 1.05 to 1.11) groups.*

We have highlighted these statistics in the abstract for simplicity and consistency with the summary (adjusted) results reported in the abstract for Covid-19 vaccine uptake. For clarity, we have also edited the statement in the results section to report the groups with the widest inequalities in both the adjusted and stratified analysis as follows:

Line 303*: Considering each vaccine eligibility group, inequalities were widest for the White and Black African (HR range 0.45 (95%ci 0.28 to 0.75) to 0.76 (95%ci 0.67 to 0.85)) and Black African (HR range 0.53 (95%ci 0.46 to 0.62) to 0.77 (95%ci 0.47 to 0.80)) groups. Following adjustment by eligibility group, overall inequalities in Influenza vaccine uptake were widest amongst White and Black Caribbean (HR 0.63 (95%ci 0.58 to 0.68) and White and Black African (HR 0.67 95%ci 0.63 to 0.72) groups (Fig 1C & Table S5).*

Please trim the limitations in your abstract (lines 30-33) down to one summarising sentence.

We have cut this to just highlight the most important limitation. It now reads as follows:

Line 32: *A limitation of this study is the focus on uptake of first doses of Covid-19 vaccination, rather than full Covid-19 vaccination.*

Lines 177 and 225: Please name and cite items located in your supplementary materials per our guidance (<https://journals.plos.org/plosmedicine/s/supporting-information>) e.g. (S1 Analysis Plan).

We have re-named supplementary materials in line with the guidance and updated the supplementary materials legends (line 580) as follows:

***S1 Appendix. Supplementary figures S1 – S5 and supplementary tables S1 – S9.***

***S2 Missing data for ethnic group. Supplementary figures S6 – S7 and supplementary tables S10 – S14.***

***S3 Analysis plan. Prospective analysis plan with information about changes made.***

***S4 STROBE checklist. Strengthening the Reporting of Observational Studies in Epidemiology (STROBE) guideline (S1 Checklist)*** *completed with section and paragraph numbers for each item.*

We have also updated citations in the main text as follows:

Line 178: *This is provided as in supplementary file ‘S3 Analysis Plan’, along with an explanation of the changes we made.*

Line 225: *Sensitivity to missing ethnicity data is explored in supplementary file ‘S2 Missing data for ethnic group’.*

Please remove the data sharing agreement and data availability section from the end of the main text as this information is captured in your Data Availability Statement as part of the submission form and will be published as metadata.

We have deleted this section (lines 471-478)

Lines 203 and 420: The terms gender and sex are not interchangeable (as discussed in <http://www.who.int/gender/whatisgender/en/> ); please ensure you are using the appropriate term in the main text and the relevant figures/tables in the supplementary materials.

We have used the term gender throughout as we consider vaccine uptake to be a predominantly socially-influenced rather than biologically-determined phenomenon. We have edited Table S10 (in S2 Missing data for ethnic group), replacing ‘sex’ with ‘gender’ for consistency.

Please review your reference list to ensure that it is complete and correct.

We have checked all references and have updated the URL for reference 16:

Department of Health & Social Care - Public Health England. The national flu immunisation programme 2019/20. 2019 [cited 26 Jan 2022] Available: https://www.england.nhs.uk/wp-content/uploads/2019/03/annual-national-flu-programme-2019-to-2020-1.pdf
